# FOXC2 promotes vasculogenic mimicry and resistance to anti-angiogenic therapy

**DOI:** 10.1016/j.celrep.2023.112791

**Published:** 2023-07-26

**Authors:** Ian G. Cannell, Kirsty Sawicka, Isabella Pearsall, Sophia A. Wild, Lauren Deighton, Sarah M. Pearsall, Giulia Lerda, Fadwa Joud, Showkhin Khan, Alejandra Bruna, Kathryn L. Simpson, Claire M. Mulvey, Fiona Nugent, Fatime Qosaj, Dario Bressan, CRUK IMAXT Grand Challenge Team, Caroline Dive, Carlos Caldas, Gregory J. Hannon

**Affiliations:** 1 Cancer Research UK Cambridge Institute, University of Cambridge, Li Ka Shing Centre, Robinson Way, Cambridge. CB2 0RE. UK; 2 New York Genome Center, 101 Avenue of the Americas, New York. NY. 10013. USA; 3 Cancer Research UK Cancer Biomarker Centre, Manchester, M20 4BX and CRUK Manchester Institute, Manchester, M20 4BX; 4 Preclinical Modelling of Paediatric Cancer Evolution team, The Institute of Cancer Research, Cotswold Road, Sutton, Surrey, SM2 5N, UK; 5 CRUK IMAXT Grand Challenge Team; 6 Department of Oncology and Breast Cancer Programme, CRUK Cambridge Centre, Cambridge University Hospitals NHS and University of Cambridge, CB2 2QQ, UK

## Abstract

Vasculogenic mimicry (VM) describes the formation of pseudo blood vessels constructed of tumor cells that have acquired endothelial-like properties. VM channels endow the tumor with a tumor-derived vascular system that directly connects to host blood vessels, and their presence is generally associated with poor patient prognosis. Here we show that the transcription factor, Foxc2, promotes VM in diverse solid tumor-types by driving ectopic expression of endothelial genes in tumor cells, a process which is stimulated by hypoxia. VM-proficient tumors are resistant to anti-angiogenic therapy and suppression of Foxc2 augments response. This work establishes co-option of an embryonic endothelial transcription factor by tumor cells as a key mechanism driving VM proclivity and motivates the search for VM-inhibitory agents that could form the basis of combination therapies with anti-angiogenics.

## Introduction

Tumors require oxygen, nutrients, and other factors supplied by the blood stream to thrive and spread. Therefore, cancer cells often release factors that stimulate angiogenesis^1–3^, a process of new host blood vessel growth. In the 1970s it was proposed that inhibition of angiogenesis could starve the tumor of essential nutrients and oxygen^2^, leading many pharmaceutical companies to develop drugs targeting pro-angiogenic factors such as vascular endothelial growth factor (VEGF). However, clinical trials of anti-angiogenic agents, such as Bevacizumab (an anti-VEGF antibody), have been largely disappointing, with most patients showing transient responses followed by inevitable resistance^3^. In retrospect, this is perhaps not surprising, as we now appreciate that tumor cells can utilize alternative mechanisms to supply themselves with necessary nutrients^4^. One such mechanism relies on vasculature formed by tumor cells themselves that adopt endothelial-like character to form extracellular matrix (ECM)-rich tubular structures that act as pseudo blood vessels, a process termed vasculogenic mimicry (VM)^5^. VM resembles embryonic vasculogenesis and since its discovery in 1999^5^ and has since been documented in multiple tumor-types, where it is almost universally a poor prognostic indicator^6^. Despite compelling evidence for the existence of VM in aggressive tumors, the molecular mechanisms driving this remarkable phenotypic transformation of cancer cells have remained elusive.

Recently, using genetic tracking of clonal lineages derived from the same parental population we identified sub-clones of mouse mammary carcinoma cell line 4T1 that are VM-competent (4T1-E^VM^ and 4T1-T^VM^)^7^. Here, we exploit these, and their VM-incompetent siblings, to identify the transcription factor FOXC2 as a key driver of vasculogenic mimicry that promotes ectopic expression of endothelial genes in tumor cells. Gene expression analysis of established cell lines, patient-derived tumor xenografts (PDTXs), and patient tumors suggest that FOXC2-driven VM is prevalent in aggressive sub-types of many solid tumors regardless of tissue of origin. Building on the work of others^5,8^, our single cell RNA-Seq (scRNA-Seq) analyses indicate that exposure to hypoxia *in vivo* promotes expression of the Foxc2 transcriptional program and acquisition of endothelial-like state in tumor cells. Importantly, loss of function experiments *in vivo* revealed a critical role for Foxc2-driven VM in resistance to anti-angiogenic therapy (AAT), suggesting possible routes to augment response to these agents.

## Results

### Visualization of perfused VM channels *in vivo* by 3D imaging

**Figure 1:**
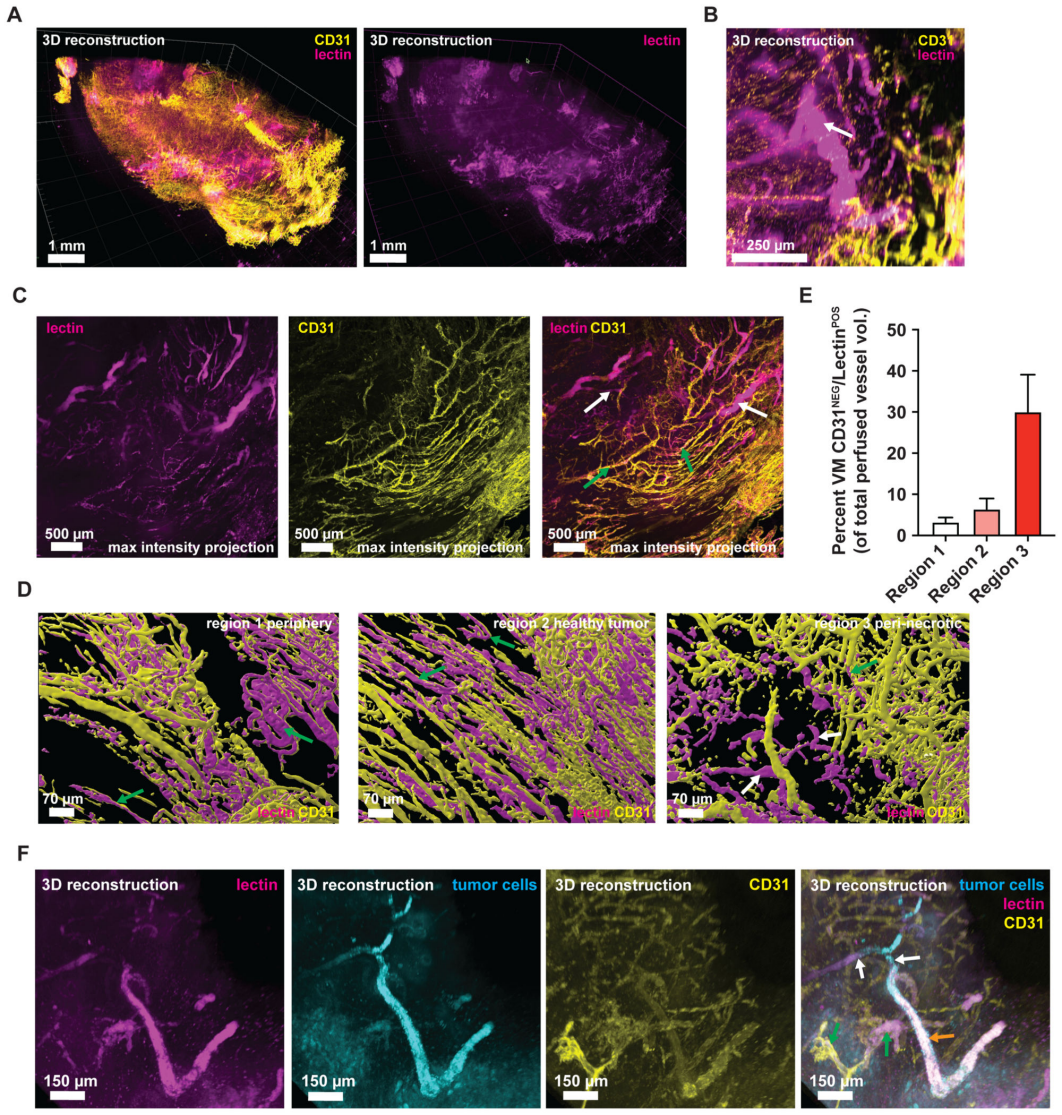
Visualization of perfused vasculogenic mimicry channels *in vivo* with lectin labelling, tissue clearing and 3D imaging. **(A)** 3D reconstruction of a ~1mm cleared 4T1-T tumor slice labelled intravenously by injection of lectin (magenta, 1A-F) and with CD31 antibody staining (yellow, 1A-F) using light sheet microscopy. **(B)** A representative lectin^POS^/CD31^NEG^ VM vessel in 3D. **(C)** Maximum intensity projections of the ~1mm Z-stack split by channel showing lectin and CD31. White arrows indicate VM vessels (lectin^POS^/CD31^NEG^), green arrows indicate host vessels (lectin^POS^/CD31^POS^). **(D)** Representative 3D renderings of CD31 and lectin in regions used for quantifying VM vessel volume. Arrows as in C. **(E)** Quantification of VM vessels in different tumor regions. The sum of CD31^POS^/lectin^POS^ (host) and CD31^NEG^/lectin^POS^ (VM) vessel volumes was calculated and the data expressed as the percent of that total that is VM. Bars mean +/- SEM, n=2 sub-regions per region. **(F)** Visualization of lectin perfusion status, CD31 and zsGreen-labelled tumor cells (cyan) of cleared 4T1-T tumors. White arrows indicate VM (lectin^POS^/CD31^NEG^/zsGreen^POS^), orange arrow indicates segments that are lectin^POS^/CD31^POS^/zsGreen^POS^ and green arrow indicates host vessel (lectin^POS^/CD31^POS^/zsGreen^NEG^).

**Figure 2:**
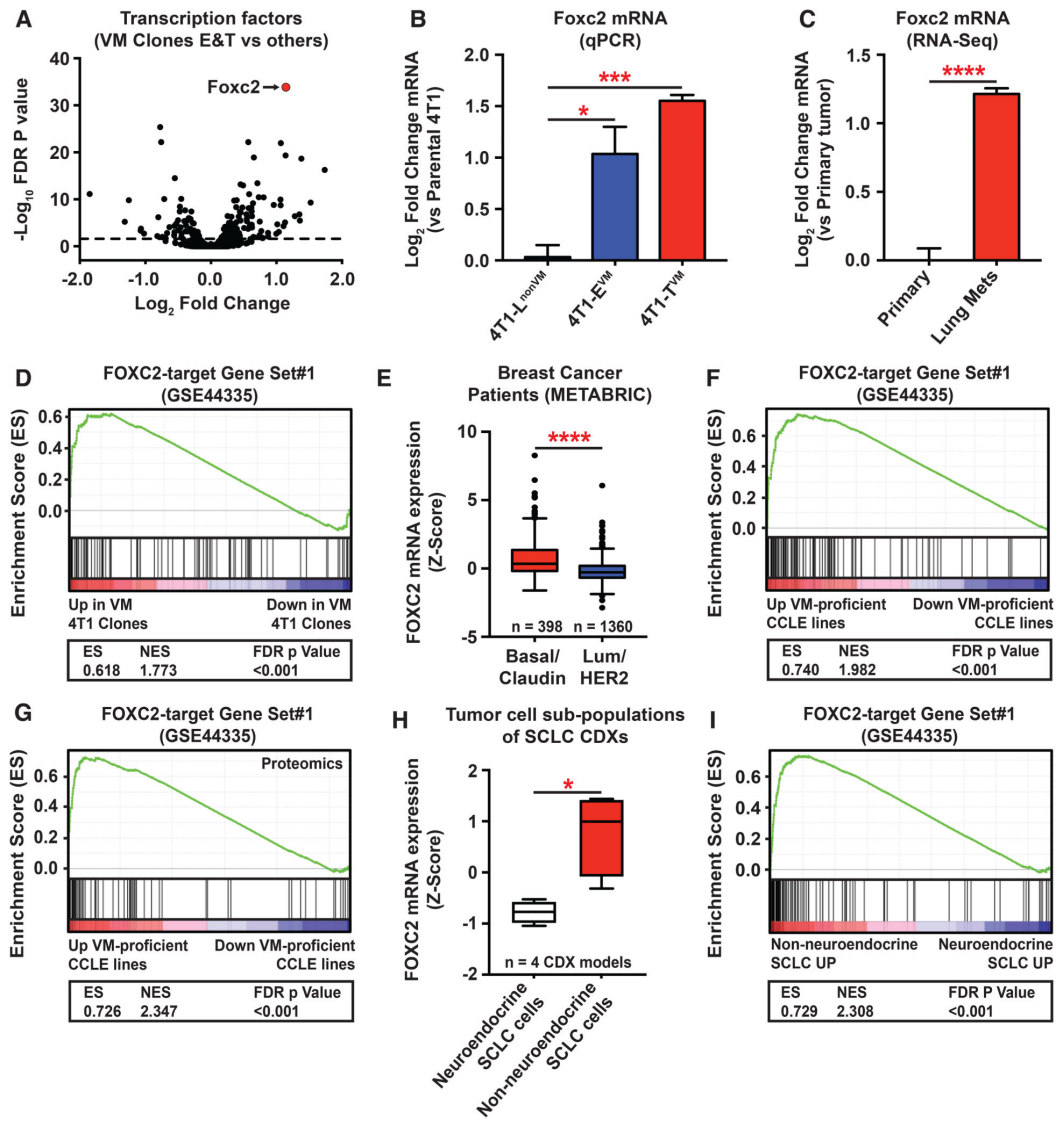
FOXC2 is up-regulated in vasculogenic mimicry-proficient tumor cells. **(A)** Volcano plot of log_2_ fold change mRNA expression vs FDR p-value of all TFs in 4T1-E^VM^ and 4T1-T^VM^, relative to all 23 4T1 sub-clones. **(B)** qRT-PCR analysis of Foxc2 mRNA expression in a subset of 4T1 sub-clones expressed as log_2_ fold change relative to parental 4T1 cells. Bars mean (+/- SEM), n=3. * p<0.05, *** p<0.001 student’s t-test. **(C)** Foxc2 mRNA expression in matched primary breast tumors and lung metastases from the 4T1 model. Bars mean (+/- SEM) log_2_ fold change vs the primary tumor, n=4. **** p<0.0001 student’s t-test. **(D)** GSEA of gene expression changes (log_2_ FC ranked) in 4T1-E^VM^ and 4T1-T^VM^, relative to all 23 4T1 sub-clones, for FOXC2-target Gene Set#1 (Table S1). ES = enrichment score, NES = normalized ES. **(E)** FOXC2 mRNA expression in breast cancer patients (METABRIC) stratified by molecular sub-type (Claudin-low/Basal vs Luminal A/Luminal B/HER2-enriched). Box plots according to the Tukey convention. n represents an individual patient. **** p<0.0001, Wilcoxon rank-sum. **(F)** GSEA as in D in VM-proficient solid tumor human cell lines (HCC38, MDA-MB-231, U87MG, NCIH446, U251MG) from the CCLE relative to all solid tumor cell lines in the CCLE, for FOXC2-target Gene Set#1. **(G)** GSEA as in F of protein expression changes (for which proteomics data exist). **(H)** FOXC2 mRNA expression from *ex vivo* cultures of SCLC circulating tumor cell-derived explants (CDX) separated into VM-competent (non-neuroendocrine) and VM-deficient (neuroendocrine) cells across multiple CDX models (CDX17, CDX17P, CDX30, CDX31P) from RNA-Seq data in Pearsall et al.,^13^. Z-scored gene expression values from paired neuroendocrine and non-neuroendocrine cells across 4 CDX models. Box plots according to the Tukey convention. n represents an individual CDX model. * p<0.05, Wilcoxon rank-sum. **(I)** GSEA as in D in non-neuroendocrine versus neuroendocrine cells derived from 4 CDX models (RNA-Seq data from H).

A longstanding controversy regarding VM is whether tumor cell-derived channels can perfuse the tumor tissue via blood transport^9^. To investigate this, we coupled intravenous administration of fluorescent Lectin with tissue clearing, CD31 antibody staining, and 3D light sheet microscopy of our 4T1-T VM-proficient tumors. This revealed a network of endothelial vasculature (yellow) (Fig 1A) that was co-labelled with lectin (magenta) (Fig 1A) and numerous CD31-negative, lectin-positive channels which we identified as vasculogenic mimics (Fig 1A), appearing throughout the tumor but enriched in peri-necrotic regions. 3D reconstruction of a lectin-positive/CD31-negative VM vessel (Figure 1B) and 2D maximum intensity projections clearly demonstrate the presence of perfused lectin-positive/CD31-negative VM vessels (Fig 1C, white arrows) and their perfused host counterparts (lectin-positive/CD31-positive, Fig 1C, green arrow) in the same image mitigating concerns of potential staining bias of the CD31 antibody (Fig 1C). To determine the spatial distribution of VM vessels in 3D we chose specific regions of interest (ROIs, Fig S1A) that demarcate the periphery (region 1), peri-necrotic area (region 3) and an area in between these two extremes (region 2) that show good quality CD31 staining. We segmented the vessels and quantified the volume that is CD31-positive/lectin-positive (host vessels) or lectin-positive only (VM vessels) using stringent filters. These analyses (Fig 1E and Fig S1B) demonstrate that VM is rare in the tumor periphery and increases closer to the necrotic tumor core consistent with a potential role for hypoxia in promoting VM^8^. Since the lectin is introduced intravenously these VM vessels must be capable of transporting it and connect into the host vasculature. The discrete nature of the 3D structures argues against staining artefacts arising from leaky, immature tumor vasculature^9^. To confirm that CD31-negative/lectin-positive structures are surrounded by tumor cells we repeated these lectin labelling experiments with zsGreen labelled 4T1-T tumor cells (Figure 1F), showing evidence for tumor cell-lined tubular structures that are lectin perfused with large areas lacking CD31 staining. Together, these data indicate that VM vessels in 4T1-T^VM^ tumors are capable of blood transport and are present throughout the tumor with enrichment in perinecrotic regions, consistent with a potential role for hypoxia in promoting VM.

### VM-proficient tumor cells upregulate FOXC2 and its transcriptional program

We reasoned that acquisition of endothelial-like character by VM tumor cells might be driven by altered expression of transcriptional regulators. Therefore, we analyzed *in vitro* RNA-Seq data from our 4T1-derived sub-clones^7^ for the expression of all transcription factors (TFs) (Fig 2A). This revealed Foxc2 as the most significantly upregulated TF in VM-proficient sub-clones, 4T1-E^VM^ and 4T1-T^VM^, when compared to all other clones (Fig 2A). This was validated in a subset of the 4T1 sub-clones by qRT-PCR (Fig 2B). Foxc2 mRNA levels were also elevated in 4T1 cells derived from lung metastases when compared to primary tumor-derived cells (Fig 2C), consistent with prior studies implicating Foxc2 in metastatic dissemination^10^. We curated a set of FOXC2-target genes from publicly available data of FOXC2 over-expression in human mammary epithelial (HMLER) cells (FOXC2-target Gene Set#1, GSE44335^11^) and this FOXC2 transcriptional program was upregulated in 4T1 VM clones (Fig 2D). Analysis of FOXC2 mRNA levels in a large cohort of breast cancer patient samples (METABRIC^12^) revealed significantly elevated levels in the aggressive Basal/Claudin-low subtypes compared to the luminal and HER2 subtypes (Fig 2E). Moreover, FOXC2-target genes were upregulated in VM-proficient human cell lines (Fig S2A) from the Cancer Cell Line Encyclopedia (CCLE) at the mRNA (Fig 2F) and protein level (Fig 2G). Finally, FOXC2 mRNA levels were significantly upregulated in VM-proficient non-neuroendocrine (non-NE) tumor cells isolated from small cell lung cancer circulating tumor cell-derived explants (SCLC CDXs), when compared to their VM-deficient neuroendocrine counterparts (Fig 2H)^13^. Consistent with this observation the broader FOXC2 program was enriched in non-NE tumor cells (Fig 2I). Taken together these data indicate that FOXC2 and its accompanying transcriptional program are upregulated in VM-proficient tumor cells.

### FOXC2 is required for vasculogenic mimicry

**Figure 3:**
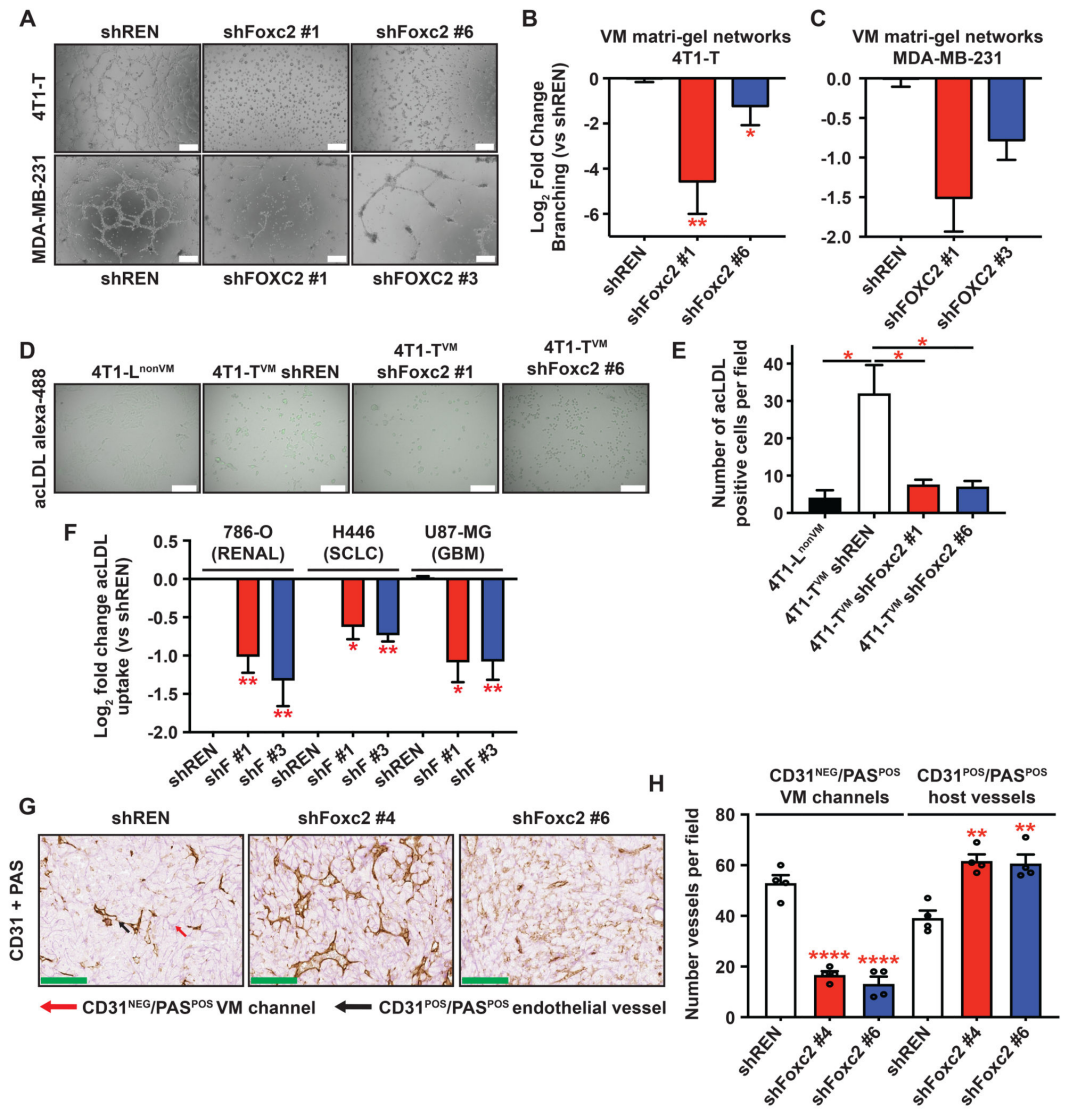
FOXC2 is required for VM and the endothelial-like properties of VM-proficient tumor cells. **(A)** Representative Matrigel network formation assay images of murine 4T1-T^VM^ or VM competent human MDA-MB-231 breast cancer cells expressing a control (shREN) or two different Foxc2-targeting shRNAs. Bar = 200 μm. **(B)** Quantification of replicate experiments in A. Bars mean log_2_ fold change (+/- SEM) in branching length vs shREN normalized for viability. n=3. * p<0.05, ** p<0.01 vs shREN, student’s t-test. **(C)** As in B with MDA-MB-231 cells. n=2. **(D)** Representative fluorescent images of Alexa-488 labelled acLDL uptake assays in 4T1-L^nonVM^ or 4T1-T^VM^ cells expressing shREN or two different Foxc2-targeting shRNAs. Bar = 200 μm. **(E)** Quantification of acLDL uptake from D. Bars mean acLDL^POS^ cells per field (+/-SEM), n=3, p<0.05, student’s t-test. **(F)** Quantification of acLDL uptake in renal, SCLC and GBM cell lines from Figure S3G. Bars mean log_2_ fold change of acLDL positive tdTomato co-positive cells relative to shREN (+/- SEM), n=3, * p<0.05, ** p<0.01, student’s t-test. **(G)** Representative images of CD31/PAS dual staining of sections from 4T1-T^VM^ tumors expressing shREN or two different Foxc2-targeting shRNAs (dox-inducible). Black arrows, endothelial vessels (CD31^POS^/PAS^POS^) and red arrows, VM vessels (CD31^NEG^/PAS^POS^). Bar = 100 μm. **(H)** Quantification of VM and host vessels from sections stained in G. Bars mean channel number (+/- SEM) per field, n=4 fields per condition from two different animals, ** p<0.01, **** p<0.0001, student’s t-test.

Given that VM appears to mimic embryonic vasculogenesis, it was notable that Foxc2 is critical for normal embryonic endothelium development^14^. Foxc2 can specify gene expression toward the endothelium^15,16^, and reprogramming of fibroblasts into endothelial-like cells requires up-regulation of endogenous FOXC2^17^, suggesting a critical role in trans-differentiation of non-endothelial cell types towards endothelium. Therefore, we asked whether Foxc2 was required for VM using a variety of assays of endothelial character. VM-proficient tumor cells, such 4T1-T^VM^, 4T1-E^VM^ ^7^, MDA-MB-231, and HCC38 (Fig S2A) form endothelial-like networks when plated on Matrigel, and performance of cells in this assay shows excellent concordance with the prevalence of CD31^NEG^/PAS^POS^ staining patterns, which are used to identify VM channels *in vivo*^5,7,18^. Knock-down of Foxc2/FOXC2 in murine mammary carcinoma 4T1-T^VM^ cells (Fig 3A, 3B and S3A) and human MDA-MB-231 claudin-low breast cancer cells (Fig 3A, 3C and S3B), with two different shRNAs, consistently suppressed network formation. FOXC2 knockdown also suppressed VM network formation in the SCLC non-NE cells from CDX17 short-term cultures (Fig S3C and S3D^13^) suggesting that the effects of FOXC2 are not restricted to established cell lines.

*Bone fide* endothelial cells, and a small subset of macrophages, have the unique and specific ability to uptake acetylated low-density lipoprotein (acLDL)^19^. Fluorescently labelled acLDL is used to isolate endothelial cells and confirm the presence of endothelial-like cells in stem cell reprograming experiments^17^. VM-proficient 4T1-Ts but not VM-deficient 4T1-Ls uptake fluorescently labelled acLDL (Fig 3D and 3E), albeit to a lesser extent than human umbilical vein endothelial cells (HUVECs) (Fig S3E). Importantly, acLDL uptake by 4T1-T^VM^ cells was diminished by Foxc2 knockdown, comparable to levels in VM-deficient cells (Fig 3D-E), and increased by Foxc2 over-expression in parental 4T1 (Fig S3F). Moreover, FOXC2 knockdown significantly impeded acLDL uptake in VM-proficient cell lines 786-O (Renal)^20^, H446 (SCLC)^18^ and U87-MG (Glioma)^21^ (Fig 3F and S3G), suggesting that the effects of Foxc2 are not restricted to breast cancer cell lines.

To assess whether Foxc2 was required for VM *in vivo* we orthotopically transplanted BALB/c mice with 4T1-T^VM^ cells transduced with a doxycycline-inducible control shRNA (shREN) or two different Foxc2-targeting shRNAs, allowing initial engraftment prior to Foxc2 knockdown. We harvested tumors 24 days post-transplantation and subjected tumor sections to CD31/PAS staining (Fig 3G), a method used to identify VM channels *in vivo* based upon the following criteria: **1**. Vessel-like morphology **2**. Periodic Acid Schiff (PAS) positivity, a basement membrane/extracellular matrix (ECM) stain, and **3**. Lack of CD31 staining, indicating they are not derived from host endothelial cells (annotated example of CD31/PAS scoring in Fig S3H)^4,5^. CD31/PAS analysis revealed a 3-4-fold reduction in the number of VM channels (CD31^NEG^/PAS^POS^) upon Foxc2 knockdown (Fig 3G and 3H), indicating that Foxc2 is required for VM *in vivo*, whilst the number of CD31^POS^/PAS^POS^ endothelial vessels increased (Fig 3G and 3H) suggesting an increase in angiogenesis and a potential crosstalk between these two tumor vascularization modes.

### Foxc2 drives ectopic expression of endothelial genes in aggressive tumor cells

**Figure 4:**
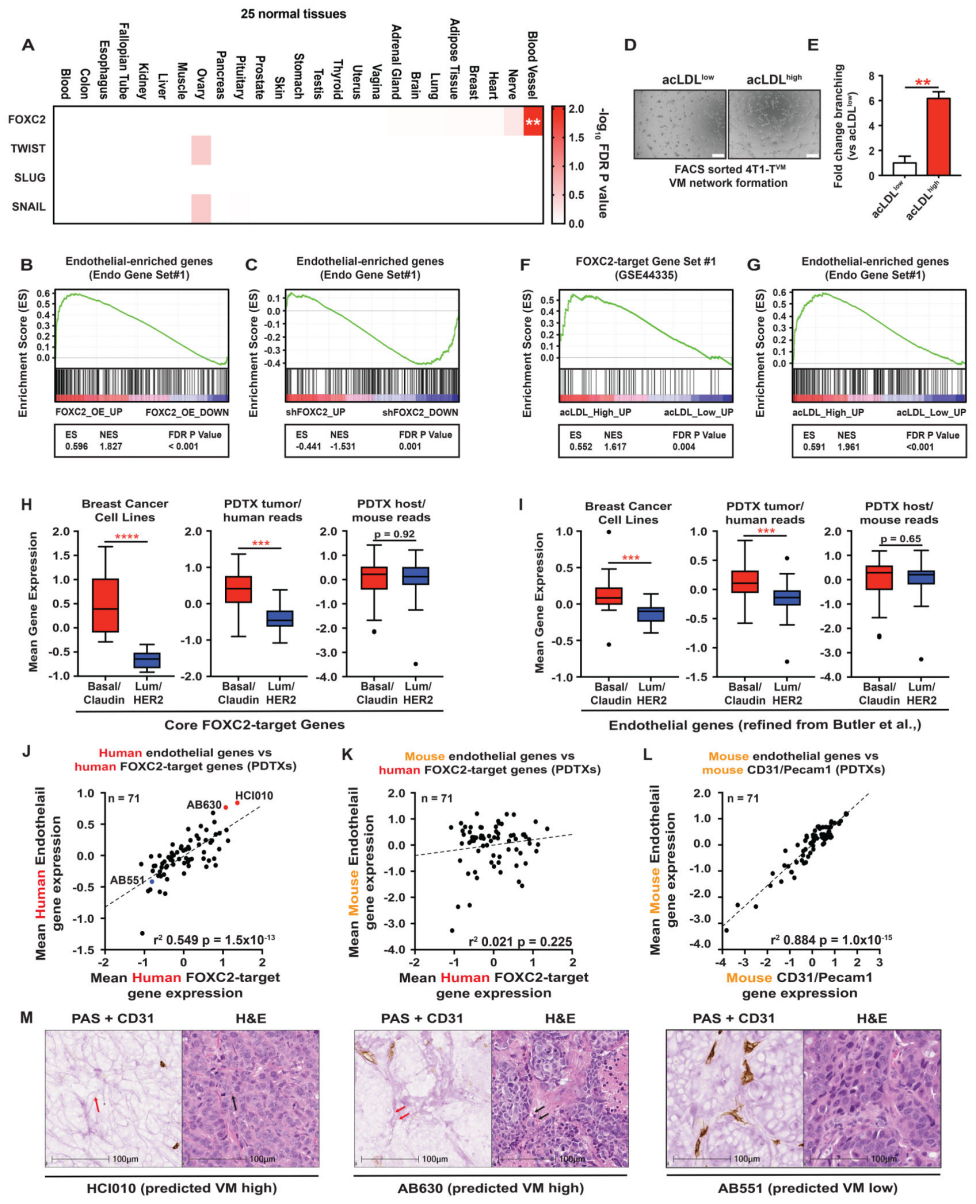
FOXC2 promotes expression of endothelial genes in tumor cells of aggressive breast cancer subtypes. **(A)** Tissue-specific expression analysis (TSEA) of top 100 up-regulated genes in HMLER cells over expressing FOXC2 (GSE4435) or the EMT transcription factors TWIST, SLUG or SNAIL (GSE43495). Color represents the -log_10_ of the Benjamini-Hochberg corrected FDR enrichment p-value for each tissue, TSEA specificity threshold of 0.001. For all gene sets see Table S1. **(B)** GSEA of gene expression changes (log_2_ FC ranked) with FOXC2 over-expression in HMLER (GSE44335) for endothelial enriched genes from Butler et al., filtered to remove any mesenchymal genes (Endo Gene Set#1, Table S1). **(C)** GSEA as in B with FOXC2 knockdown in MDA-MB-231 human breast cancer cells. **(D)** Representative Matrigel network formation assays of 4T1-T^VM^ cells sorted based on high or low acLDL uptake. Bar = 200 μm. **(E)** Quantification of Matrigel network formation assays in D. Bars mean fold-change (+/- SEM) in branching length vs the acLDL^low^ population. n=3, ** p<0.01, student’s t-test. **(F)** GSEA as in B. RNA-seq of acLDL^high^ and acLDL^low^ 4T1-T cells sorted as in D using FOXC2-target Gene Set#1. **(G)** GSEA as in F using Endo Gene Set#1. **(H)** Expression of core FOXC2-target genes (FOXC2-target Gene Set#3) in human breast cancer cell lines from the CCLE, PDTX tumor cells (human reads), or PDTX stroma/host cells (mouse reads). Box plots according to the Tukey convention. Mean signature expression was calculated for each cell line or PDTX, n represents an individual cell line or PDTX model. *** p<0.001, **** p<0.0001, Wilcoxon rank-sum. **(I)** As in H for endothelial enriched genes (Endo Gene Set#1). **(J)** Pearson correlation between tumor/human Endo Gene Set#1 expression and tumor/human FOXC2-target Gene Set#3 expression across PDTXs. Mean signature expression was calculated as in H and I. n=71 models, red = VM-high, blue = VM-low. **(K)** Pearson correlation between host/mouse endothelial gene expression (Endo Gene Set#1, mouse orthologs) and tumor/human FOXC2-target Gene Set#3 expression across PDTXs. Mean signature expression was calculated as in H and I. n=71 models. **(L)** Pearson correlation between host/mouse endothelial gene expression (Endo Gene Set#1, mouse orthologs) and mouse Pecam1 (encoding CD31) gene expression across PDTXs. Mean signature expression was calculated as in H and I. n=71 models. **(M)** Representative images of CD31/PAS staining with adjacent H&E staining from three PDTXs. HCI010 and AB630, predicted VM-high (red dots, Figure 4J) and AB551, predicted VM-low (blue dot, Figure 4J). Red arrows indicate PAS^POS^/CD31^NEG^ channels with red blood cells on the adjacent H&E section. Bar = 100 μm.

To explore the genes that FOXC2 regulates to drive VM, we analyzed publicly available data from FOXC2 over-expressing HMLER cells (GSE44335)^11^ by tissue specific-expression analysis (TSEA, http://genetics.wustl.edu/jdlab/tsea). This analysis revealed significant (p=0.009) and specific enrichment of FOXC2-targets for blood vessel-specific genes (Fig 4A). Since FOXC2 has been previously implicated in epithelial to mesenchymal transition (EMT)^10,11^ we compared the TSEA profile of FOXC2-targets to that of canonical EMT transcription factors^22^ and observed no significant enrichments (Fig 4A), suggesting that FOXC2 is unique among EMT TFs in driving expression of vascular-specific genes in non-endothelial cells. Moreover, global analysis of gene expression changes upon over-expression of FOXC2 or EMT TFs revealed strong correlations between TWIST, SLUG and SNAIL but a distinct lack of correlation with FOXC2-induced changes (Fig S4A) indicating that FOXC2 controls a gene expression program distinct from canonical EMT transcription factors. To further explore this, we analyzed expression of endothelial genes in HMLER cells over expressing FOXC2 or MDA-MB-231 cells with knockdown (RNA-Seq data generated for this study) using GSEA and an endothelial gene set from Butler et al^23^, refined to remove mesenchymal genes^24^ (hereafter named Endo Gene Set#1). This analysis revealed upregulation of endothelial genes with FOXC2 over-expression (Fig 4B) and downregulation with knockdown (Fig 4C). Gene Ontology analysis^25^ revealed downregulation of genes involved in ECM organization, secretion, hypoxia response, and regulation of vasculature development upon FOXC2 knockdown in MDA-MB-231 cells (Fig S4B), mirroring our observations with over-expression using TSEA.

To ascertain whether tumor cell endothelial gene expression was directly linked to functional endothelial-like properties we flow sorted 4T1-T^VM^ cells based on differential acLDL uptake and profiled them by RNA-Seq. VM Matrigel network formation assays demonstrated that acLDL^high^ cells are competent to form networks (Fig 4D and 4E) and are enriched for FOXC2-target and endothelial genes (Fig 4F and 4G) orthogonally indicating that FOXC2 transcriptional activity, endothelial gene expression, network formation and acLDL uptake are all *ex vivo* measures of the same biological process, presumably VM propensity.

To explore FOXC2-driven VM in human tumors and patient-derived models we defined a set of 5 core FOXC2-target genes (FOXC2-target Gene Set#3, Table S1, Fig S4E and methods). Analysis of human breast cancer cell lines from the CCLE, stratified by molecular subtype, revealed significantly elevated expression of these FOXC2-target genes (Fig 4H), endothelial genes (Fig 4I) and proteins (Fig S4F) in the Basal/Claudin-low subtypes compared to luminal subtypes. We next sought to determine whether these enrichments were also evident in models that more faithfully recapitulate patients’ tumors. We analyzed RNA-Seq data derived from a collection of molecularly annotated breast cancer PDTXs^26,27^ where reads were mapped to a combined human and mouse reference genome allowing us to deconvolve gene expression arising from the human/tumor and mouse/stromal compartments^28^. This analysis revealed elevated expression of FOXC2-target genes (Fig 4H) and endothelial genes (Fig 4I) in aggressive Basal/Claudin-low tumors only in the human/tumor compartment and not the mouse/stromal compartment (Fig 4H and 4I). Further analysis of the PDTX human gene expression data demonstrated a strong correlation between human FOXC2-target genes and human endothelial genes across all PDTX models (Fig 4J). This relationship was not maintained when comparing expression of human FOXC2-target genes with mouse endothelial genes (Fig 4K), whereas a strong correlation was observed between expression of Pecam1 (murine CD31) and mouse endothelial genes (Fig 4L), robustly validating our endothelial gene signature and species mapping deconvolution of tumor/stroma approach.

To determine FOXC2 protein levels in PDTXs we performed immunofluorescence for FOXC2 on 7 models (Fig S4G) and derived an H-Score using HALO software. The IF-derived H-Score strongly correlated with human FOXC2-target Gene Set#3 expression (Fig S4H, r^2^=0.902 p=0.0011) and human endothelial gene expression (Endo Gene Set#1) (Fig S4I, r^2^=0.932 p=0.0004) but not with mouse endothelial genes (Fig S4J). These data support the use of core FOXC2-target genes as surrogates of FOXC2 activity in available gene expression data lacking protein measurements, and orthogonally confirm the strong relationship between FOXC2 and ectopic endothelial gene expression in tumors. FOXC2-target genes also correlated with endothelial genes across breast cancer patient tumors from the METABRIC cohort (Fig S4K), though determining the relative contribution of tumor and stroma to this correlation is not possible. The existence of archival PDTX tissue enabled us to test our gene expression predictions of VM proclivity using CD31/PAS staining. We stained sections from two PDTXs predicted to be VM-high based on gene expression (HCI010 and AB630) and one predicted to be VM-low (AB551) (Fig 4M). Both models predicted to be VM-high showed extensive areas of PAS^POS^ structures with vessel-like morphology lacking CD31 expression (Fig 4M), consistent with high levels of VM. Importantly, several of these PAS^POS^/CD31^NEG^ channels contained red blood cells within an adjacent H&E section (Fig 4M, black arrows). Conversely, in AB551, predicted to be VM-low, the PAS^POS^ vessel-like structures were predominantly CD31^POS^ consistent with the presence of host derived vasculature (Fig 4M). Analysis of publicly available gene expression data from several other solid tumor types showed elevated levels of FOXC2-target genes in aggressive subtypes of colorectal cancer, glioblastoma and ovarian carcinoma (Fig S4L). Our core FOXC2-target genes correlate with endothelial genes across cell lines from diverse tumor types and patient samples (Fig S4M) and stratify survival in CMS4 colorectal cancer, mesenchymal ovarian cancer, and renal cancer (Fig S4N). Together, these cell line, patient and PDTX data indicate that VM is enriched in the aggressive subtypes of human tumors and that combined tumor-endothelial and core FOXC2-target gene expression can identify VM proficient tumors.

### Foxc2 links hypoxia with the acquisition of VM capabilities

**Figure 5:**
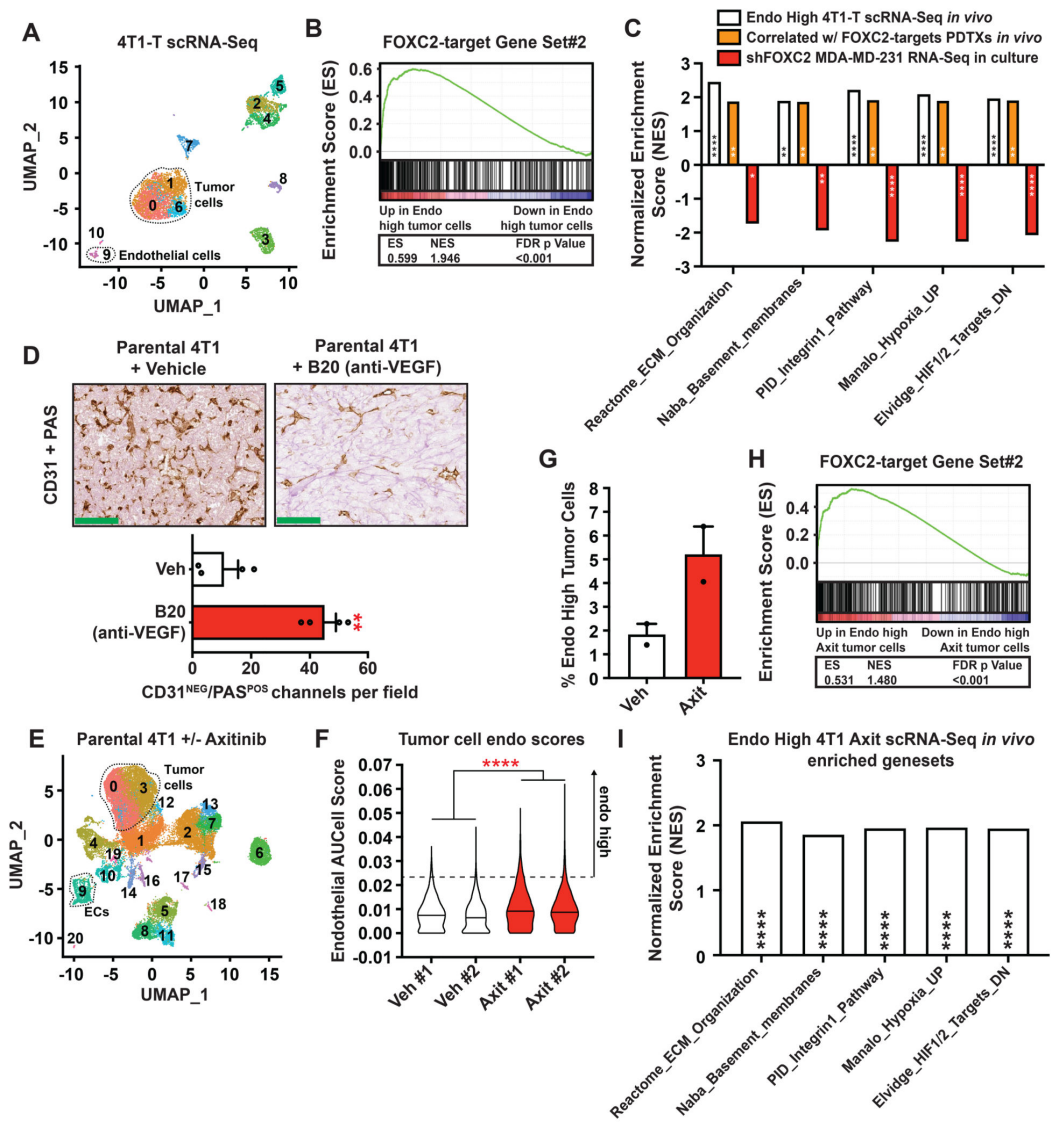
Severe hypoxia promotes quasi-endothelial differentiation of tumor cells. **(A)** UMAP visualization of 4T1-T^VM^ CellTag tumor scRNA-seq data, labelled by cluster number with tumor cells and endothelial cells highlighted. **(B)** GSEA gene expression changes in endo-high 4T1-T tumor cells vs the remaining tumor cells for FOXC2-target Gene Set#3. Endo-high = top 5% of cells based on expression of the Endo Gene Set#2. **(C)** GSEA-derived normalized enrichment scores (NES) of select gene sets across datasets consisting of the endo-high scRNA-Seq ranked list (in 5B), human genes ranked based on their correlation with human FOXC2-target genes across PDTXs and genes ranked based on their log_2_ fold change with FOXC2 knockdown in MDA-MB-231 cells. * p<0.05, ** p<0.01, **** p<0.0001, GSEA-derived FDR p value. **(D)** Representative images of CD31/PAS staining of parental tumors treated with B20. Bar = 100 μm. Below quantification of CD31^NEG^/PAS^POS^ channels in B20 treated parental tumors. Bars mean number of channels (+/- SEM) per field, n=4 fields per condition from two different animals, ** p<0.01, student’s t-test. For quantification of host vessels and VM ratios see Figure S5I. **(E)** UMAP visualization of parental 4T1 CellTag tumor scRNA-seq dataset as in 5A +/- Axitinib. **(F)** Distribution of AUCell-calculated endo scores (Endo Gene Set#2) of all tumor cells separated by replicate and treatment. **** p<0.0001, Wilcoxon rank-sum comparing vehicle to Axitinib treatment. **(G)** Percentage of endo-high tumor cells based on AUCell scores in F (above the line). Bars represent mean percentage of endo-high cells, n=2 animals per treatment condition. **(H)** GSEA of gene expression changes in endo-high 4T1 Axitinib treated parental tumor cells vs the remaining Axitinib treated parental tumor cells for FOXC2-target Gene Set#3. **(I)** GSEA-derived NES for select gene sets using the ranked list from H as input. The same gene sets are highlighted in C. **** p<0.0001, GSEA-derived FDR p-value.

To further explore the relationship between tumor cell endothelial gene expression and Foxc2 *in vivo* we utilized scRNA-Seq of 4T1-T^VM^ cells labelled with CellTag^29^, which encodes a GFP transcript that can be captured by standard 10X scRNA-Seq workflows enabling computational identification of tumor cells (Fig S5A). Figure 5A shows a broad overview of cell types present within these tumors and the identity of the endothelial and tumor cell clusters. Expression of classical endothelial genes in different cell types within the dataset (Figure S5B) highlights a subset of tumor cells with high expression of these genes. To systematically define such cells within our data we further refined the endothelial gene set from Butler et al.,^23^ by performing differential expression between fibroblasts and endothelial cells in our scRNA-Seq data (from parental 4T1 tumors), removing any genes expressed in fibroblasts. With this highly stringent set of endothelial genes (Endo Gene Set#2, Table S1), we calculated an endo-score for each individual tumor cell from our 4T1-T^VM^ scRNA-Seq data using AUCell and performed differential expression between endo-high cells (top 5% of cells based on their endo-score) with the remaining tumor cells. GSEA confirmed enrichment of a stringent FOXC2-target gene set (FOXC2-target Gene Set#2, Table S1) in endo-high cells (Fig 5B). GSEA also revealed enrichment of ECM and hypoxia-related signatures in endo-high cells *in vivo* (Fig 5C), that were also enriched in genes that correlate with FOXC2-targets across PDTXs and genes downregulated by FOXC2 knockdown *in vitro* (Fig 5C). Importantly, Pearsall et al.,^13^ also observe ECM and hypoxia signatures enriched within VM-competent non-NE SCLC cells and show a functional role for integrin b1 (“integrin1 pathway” is a top enriched gene set) in promoting VM network formation. Our gene expression data combined with the prior work of others suggest that hypoxia may act as a trigger for VM *in vivo* via FOXC2^8^. As such, exposure of non-VM parental 4T1 cells to severe hypoxia (0.1% O_2_) culture endowed the surviving cells with network forming abilities (Fig S5C and S5D). Since hypoxia-related gene sets dominated the GSEA results from FOXC2 knockdown MDA-MB-231 cells (Fig S5F and S5G), we reasoned that FOXC2 expression may promote survival under hypoxic conditions. Consistent with this notion Foxc2-knockdown parental 4T1s were more sensitive to hypoxia-induced cell death than their control counterparts (Fig S5E) suggesting an important role for FOXC2 in hypoxia tolerance.

To investigate the role of HIF signaling in hypoxia-mediated acquisition of VM gene expression, we analyzed publicly available data (GSE42791^30^) of H460 NSCLC cells exposed to hypoxia (1% O_2_) or hypoxia plus the HIF inhibitor BAY-872243. Hypoxia led to an increase in FOXC2-target genes and endothelial genes in response to hypoxia that was blunted by concomitant HIF inhibition (Figure S5H). Analysis of HIF1a/2a-target genes and hypoxia-induced genes confirmed the expected effects of HIF inhibition (Fig S5H). These data indicate that hypoxia promotes VM via HIF and FOXC2 signalling, although the precise relationship between these two important transcription factors remains to be elucidated.

To examine hypoxia-induced VM *in vivo*, we treated parental 4T1 tumors with the VEGF blocking antibody B20-4.1.1^31^ (hereafter B20, blocks human and mouse VEGF), which promotes hypoxia by blocking angiogenesis. This treatment increased CD31^NEG^/PAS^POS^ VM channels (Fig 5D and Fig S5I) indicating that tumor cells exposed to severe hypoxia become more VM competent. To explore the connection between hypoxia and VM further and determine whether these cells acquire endothelial and Foxc2 gene expression programs, we utilized scRNA-Seq of CellTag labelled parental 4T1 tumors exposed to the VEGFR1-3 inhibitor Axitinib^32^. We clustered the entire dataset (Fig 5E and S5J), regardless of CellTag expression, and confirmed that Axitinib induced hypoxia in tumor cells (Fig S5K) and suppressed angiogenesis and proliferation in endothelial cells (Fig S5L). Tumor cells were identified based on their overall gene expression, proximity within the UMAP plot and were required to have >20% of cells within the cluster expressing CellTag (Fig S5J). Individual tumor cells were assigned an endo-score using AUCell and our stringent endothelial gene set (Endo Gene Set#2). As shown in Figure 5F, Axitinib significantly increased endo-scores in tumor cells both shifting the distribution towards higher scores overall (Fig 5F) and increasing the fraction of endo-high cells (Fig 5G). We then performed differential expression analysis between the endo-high cells (top 5%) and the remaining tumor cells within the Axitinib treated tumors. As in 4T1-T, endo-high cells within Axitinib treated tumors showed enrichment for FOXC2-targets (Fig 5H), ECM, and hypoxia gene sets (Fig 5I) indicating that exposure to hypoxia *in vivo* through AAT promotes VM and the acquisition of an ECM-producing quasi-endothelial state in tumor cells.

### FOXC2-driven VM promotes resistance to anti-angiogenic therapy

It has been postulated that VM may underlie tumor resistance to AAT^4,33^. VM vessel formation does not depend on major canonical angiogenic signals, such as the VEGF pathway, and VM network formation is indifferent to VEGF inhibition *in vitro* (Fig S6A). While VM-driven resistance to AAT is an attractive hypothesis, convincing empirical evidence supporting this notion is lacking. We therefore sought to leverage both our understanding of VM drivers and our ability to manipulate VM to examine whether VM mediates AAT resistance.

**Figure 6:**
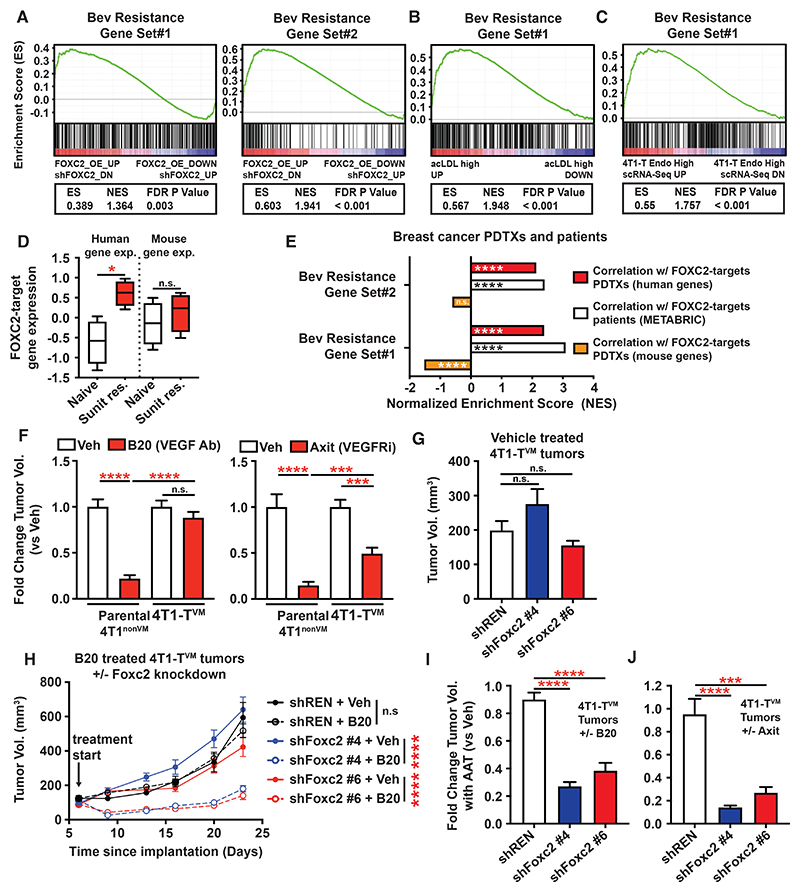
FOXC2-driven VM promotes resistance to anti-angiogenic therapy. **(A)** GSEA of gene sets associated with AAT resistance in breast cancer patients (Bev Resistance Gene Set#1) or GBM xenografts (Bev Resistance Gene Set#2) in a meta-analysis of FOXC2 driven gene expression changes. Ranked list derived from mean of the log_2_ fold change upon FOXC2 over-expression in HMLER cells and the inverse log_2_ fold change upon FOXC2 knockdown in MDA-MB-231 cells. **(B)** GSEA of AAT resistance genes from breast cancer patients (Bev Resistance Gene Set#1) in acLDL^high^ versus acLDL^low^ 4T1-T^VM^ cells, using RNA-seq data in Figure 4D and 4E. **(C)** GSEA as in B in endo-high versus bulk 4T1-T^VM^ cells using scRNA-seq data in Figure 5A and 5B. **(D)** Mean Z-score expression of FOXC2-target Gene Set#3 in naive or Sunitinib resistant renal PDX using either human (tumor) or mouse (stroma/host) microarrays (GSE76068). Box plots according to the Tukey convention. Mean Z-score expression was calculated per replicate, n represents an individual mouse. * p<0.05, Wilcoxon rank-sum test. **(E)** GSEA summary statistics of ranked lists of human FOXC2-target Gene Set#3 correlations with human/tumor genes (red bars), with mouse genes (orange bars) or with genes across patients from the METABRIC cohort (white bars), used as inputs for GSEA of AAT resistance genes. **** p<0.0001, GSEA-derived FDR, n.s. = not significant. **(F)** Tumor volumes of parental 4T1^nonVM^ or 4T1-T^VM^ tumors treated with B20 or Axitinib. Bars represent mean fold change (+/- SEM) relative to vehicle. n=10 mice per condition. *** p<0.001, **** p<0.0001, n.s. not significant, student’s t-test. **(G)** Tumor volumes of vehicle treated animals with Foxc2 knockdown 4T1-T^VM^ tumors vs control (shREN) tumors. Bars mean tumor volume (mm^3^) (+/- SEM). n.s. = not significant. **(H)** Growth curves of 4T1-T tumors treated with vehicle or B20 with or without Foxc2 knockdown by doxycycline-inducible shRNAs. Curves mean tumor volume (+/- SEM) in mm^3^ over time. Two-way ANOVA effect of treatment F (1, 18): shREN Vehicle (n=10) vs shREN B20 (n=10) = 0.001025, p-value = 0.975. shFoxc2 #4 Vehicle (n=10) vs shFoxc2 #4 B20 (n=9) = 55.13, p-value <0.0001****. shFoxc2 #6 Vehicle (n=10) vs shFoxc2 #6 B20 (n=10) = 29.19, p-value <0.0001****. **(I)** Response of shREN or Foxc2 knockdown 4T1-T^VM^ tumors to B20 (17 days). Bars mean fold change (+/- SEM) in tumor volume relative to shREN, normalized to vehicle per condition. n=10, 9, 10 mice per condition. **** p<0.0001, student’s t-test. **(J)** Response of shREN or Foxc2 knockdown 4T1-T^VM^ tumors to Axitinib (10 days) as in I. n=10, 9, 10 mice per condition. *** p<0.001, **** p<0.0001, student’s t-test.

We curated gene sets associated with resistance to Bevacizumab (an anti-VEGF antibody) in breast cancer patients^34^ or in a glioblastoma (GBM) xenograft (GSE37956)^35^ (Table S1). We then analyzed enrichment of these gene sets with GSEA using a meta-dataset of FOXC2-regulated gene expression. As shown in Fig 6A, genes associated with failure to respond to Bevacizumab in breast cancer patients (Bev Resistance Gene Set#1) or genes upregulated in GBM xenografts upon Bevacizumab resistance (Bev Resistance Gene Set#2) showed significant enrichment in FOXC2-activated genes, but not EMT-associated genes (Fig S6B), consistent with a potential role for FOXC2 in AAT resistance. To ascertain whether these enrichments were driven by effects on VM, we used our orthogonal acLDL RNA-Seq dataset (Fig 6B) and our endo-high 4T1-T scRNA-Seq dataset, finding enrichment of breast cancer (Fig 6B and 6C) and GBM (Fig S6C) AAT resistance genes in acLDL high or endo-high tumor cells, independently implicating VM in AAT resistance. Analysis of a publicly available dataset of Sunitinib resistance in a renal cancer PDX model^36^ also demonstrated up-regulation of core FOXC2-target genes specifically in the human/tumor compartment (Fig 6D) upon Sunitinib resistance. Further analysis of our breast cancer PDTXs revealed enrichment of Bev resistance genes in human, but not mouse, genes that correlate with human FOXC2-targets (Fig 6E), suggesting that the Bevacizumab resistance gene signature is of tumor origin. Similar analyses of cell lines and primary tumors of other tumor-types likewise showed enrichment of Bevacizumab resistance genes from breast cancer patients and GBM xenografts (Fig S6D). Considered together, these observations are consistent with FOXC2-driven VM being associated with failure of AAT in preclinical models and human patients.

To investigate whether VM influences AAT sensitivity, we utilized the 4T1 model. Treatment of parental 4T1^nonVM^ tumors with B20 decreased tumor volume by 75% compared to vehicle, whereas B20 treatment of 4T1-T^VM^ tumors had no significant effect (Fig 6F). Similar results were obtained when comparing response of parental 4T1^nonVM^ and 4T1-T^VM^ tumors to Axitinib (Fig 6F), albeit with a greater overall response. We reasoned that if VM was driving resistance to AAT in 4T1-T^VM^ tumors, suppression of Foxc2 might augment AAT response. To test this, we orthotopically transplanted BALB/c mice with 4T1-T^VM^ cells transduced with doxycycline-inducible shRNAs against a control sequence (shREN) or two different Foxc2 sequences, then treated with B20 or vehicle 7 days post-transplantation. Foxc2 knockdown had no significant effect on tumor growth in vehicle treated animals (Fig 6G). Control shRNA tumors showed only ~10% reduction in tumor volume in response to B20 (Fig 6H and 6I) whereas suppression of Foxc2 drastically flattened tumor growth curves upon B20 treatment (Fig 6H), reducing tumor volume ~70% relative to vehicle (Fig 6H and 6I). Similarly, while control tumors showed ~5% reduction in tumor volume with Axitinib treatment, Foxc2 knockdown tumors showed ~80% reduction in size with treatment (Fig 6J). Survival analysis of tumor bearing mice confirmed that animals with Foxc2 knockdown 4T1-T^VM^ tumors significantly benefited from B20 treatment (Fig S6E) compared to control 4T1-T^VM^ animals. Overall these mouse and human data strongly suggest that Foxc2-driven VM promotes resistance to AAT *in vivo*.

## Discussion

VM was first described over 20 years ago^5^. Its presence has been catalogued across human tumor specimens using CD31/PAS staining^6^, revealing that the frequency of CD31-negative/PAS-positive patterns correlate with reduced survival across many of tumor-types. However, VM has been a controversial area with some investigators arguing that tumor-derived non-endothelial lined blood vessels do not exist and that their observation is an artifact of thin 2D sections leading to erroneous assignment of CD31-negative status due to missing the endothelial cells in the Z plane^9^. Moreover, it has been claimed that even if such structures do exist, they are likely to lack blood carrying capacity due to lack of pericyte coverage and clotting^9^. To address these concerns we have performed intravenous injection of animals harboring VM-proficient 4T1-T tumors with fluorescently labelled lectins followed by tissue clearing and light sheet microscopy to perform 3D imaging of perfusion (lectin fluorescence) and the host vasculature by CD31 immunofluorescence. These data clearly demonstrate the presence of VM vessels within the tumor that are lectin-positive without surrounding CD31-positive endothelial cells but with surrounding tumor cells (Figure 1). Since the lectin is introduced intravenously these VM vessels must be capable of carrying blood and connect to the host vasculature. As this is volumetric imaging, it is difficult to sustain the argument that lack of CD31 staining is due to the sectioning procedure.

Despite a clear association with poor outcomes for patients^6^, a unifying underlying mechanism by which tumor cells acquire VM capabilities has been elusive. Here, we have shown that VM-proficient tumor cells from breast and SCLC, at least, up-regulate an embryonic endothelial TF FOXC2 to drive VM (Fig 2 and Fig 3). FOXC2 appears to achieve this through the transcriptional regulation of endothelial gene expression in tumor cells, leading to their acquisition of endothelial-like properties (Fig 4). Prior to our work, the most established role for FOXC2 in cancer was as an EMT regulator and in metastasis^10,11^. While our data indicates that FOXC2 promotes loss of epithelial character in tumor cells, the extent to which it endows cells with mesenchymal character is less clear. For example, mesenchymal genes are not consistently down-regulated (Fig S4C)^24^ in FOXC2 knockdown MDA-MB-231 cells. In fact, some mesenchymal markers are up-regulated (e.g. CDH2/N-Cadherin), whereas others are down-regulated (e.g. FN1/Fibronectin) (Fig S4D). Moreover, the FOXC2 transcriptional program does not correlate with that of canonical EMT TFs (Fig S4A), suggesting that FOXC2 has distinct functions. This suggests that FOXC2 confers a quasi-endothelial identity upon tumor cells that may be acquired via an intermediate mesenchymal state. This is supported by our observations in colorectal cancer patient data in which FOXC2-target genes are overall enriched in the CMS4 “mesenchymal” subtype (Fig S4L). Nonetheless, these genes are still able to further stratify CMS4 patients into a particularly poor survival sub-group (Fig S4N), suggesting that VM-competent tumors are a subset of this mesenchymal group with additional characteristics.

More than 45 years ago, Judah Folkman proposed that targeting the normal cells of the patient^2^ rather than tumor cell themselves would result in less resistance and more effective treatment. However, AATs have proved largely disappointing. What Folkman did not anticipate was the ability of tumor cells to undergo epithelial-to-endothelial transition driven by co-option of an embryonic endothelial TF FOXC2, creating tumor-derived vasculature that does not depend on canonical angiogenic signals^4,5^. Our data suggest that VM not only underlies resistance to AAT but is driven by the very scenario created by it, oxygen starvation. This provides a strong motivation to search for pharmacological inhibitors of VM that might be used in combination with AAT to starve tumors of the nutrients that they require for survival.

## Limitations of the study

VM has been a controversial idea among the vascular biology community, with few believing that tumor cell-lined vessels exist. This likely arises from CD31/PAS staining as the main approach employed to identify VM. To address this issue, we have used fluorescent lectins to label the vasculature followed by tissue clearing, host vasculature antibody staining and 3D imaging to holistically assess the functional status of vessels within putative VM tumors. This approach has demonstrated the presence of CD31-negative, tumor cell-lined, lectin perfused vessels. Making these types of measurements across many samples is technically complex and time consuming, therefore we rely on CD31/PAS staining to a certain extent. 2D measurements of the vasculature, such as CD31/PAS staining, likely misses CD31-positive vessels due to the use of thin sections and likely over-estimates VM vessels, and this should be a consideration when interpreting this assay. Conversely, lectin labelling may underestimate VM vessels since labelling is not perfectly efficient and whether all VM vessels express the requisite lectin-binding glycoproteins is unknown, although as evidenced in our data some must. In summary, the true extent of VM probably lies between the estimates derived from these two orthogonal modalities.

## STAR methods

### Resource availability

#### Lead Contact

Further information and requests for resources and reagents should be directed to and will be fulfilled by the lead contact, Greg Hannon (greg.hannon@cruk.cam.ac.uk).

#### Materials availability

Resources and reagents requests should be directed to, and will be fulfilled by, the lead contact.

#### Data and code availability

- Single-cell and bulk RNA-seq data have been deposited at GEO and are publicly available as of the date of publication under the accession numbers GSE230643 (scRNA-seq) and GSE232214 (bulk RNA-seq). Accession numbers are also listed in the key resources table. Other original data will be shared by the lead contact upon request.
- This paper does not report original code.
- Any additional information required to reanalyze the data reported in this paper is available from the lead contact upon request.

## Experimental model and study participant details

### Mice

All mouse experiments used female BALC/c mice (Charles River (strain code 028) or Taconic Biosciences (BALB/cAnNTac)) that were 6-8 weeks old and 17-22 grams at the time of implantation. Mice were housed in individually ventilated cages with wood chip bedding and nestlets with environmental enrichment (cardboard fun tunnels and chew blocks) under a 12 h light/dark cycle at 21 ± 2 °C and 55% ± 10% humidity. Diet was irradiated LabDiet 5R58 with ad libitum water unless otherwise specified. Mice were housed socially and randomly assigned to treatment groups on a per cage basis, where possible. Any animals that did not have palpable tumors at day 7 post-implantation were removed from the analysis. All mouse experiments were approved by the Rockefeller University’s institutional animal care and use committee (IACUC) or performed under the Animals (Scientific Procedures) Act 1986 in accordance with UK Home Office licenses (Project License # PAD85403A) and approved by the Cancer Research UK (CRUK) Cambridge Institute Animal Welfare and Ethical Review Board. Briefly, 50,000 parental or 4T1-T cells, resuspended in 50 μl of a 1:1 mixture of growth factor-reduced Matrigel:PBS, were injected into the 4^th^ mammary fat-pad of 6-8 week old female BALB/c mice (Taconic Biosciences or Charles River). Drug treatments were initiated once palpable tumors had formed at 6 days post-transplantation. B20-4.1.1 (a gift from Genentech) was resuspended in B20 vehicle (10 mM Histidine, 140 mM NaCl, 0.02% Tween-20) and treatment was administered by intraperitoneal injection at a final concentration of 5 mg/Kg, twice a week for a maximum of 3 weeks. Axitinib (MedChem Express, HY-10065) was resuspended in PBS supplemented with 0.5% Carboxymethylcellulose and treatment was administered by intraperitoneal injection at a final concentration of 50 mg/Kg, once a day for 5 days followed by 2 days off treatment, for a maximum of 3 weeks. For shRNA induction experiments animals were switched to doxycycline containing diet (Bioserv, S3888) 2 days post-transplantation and drug treatments were initiated 4 days later i.e. 6 days post-transplantation.

### Cell lines

Parental 4T1 (ATCC, CRL-2539, RRID:CVCL_0125) 4T1 clonal lines^7^, MDA-MB-231s (ATCC, HTB-26, RRID:CVCL_0062, Female), HCC38 (ATCC, CRL-2314, RRID:CVCL_1267, Female), NCI-H446 (ATCC, HTB-171, RRID:CVCL_1562, Male), and 786-O (ATCC, CRL-1932, RRID:CVCL_1051, Male) were grown in RPMI supplemented with 10% fetal bovine serum (FBS) and L-glutamine. U-87 MG (ATCC, HTB-14, Male), Platinum-A retroviral packaging cells (Cell Biolabs Inc, RV-102, Female), and 293 FT (Thermo Fisher Scientific, RRID:CCVCL_6911, Female) cells were grown in DMEM supplemented with 10% FBS and L-glutamine. All lines were grown at 37°C with 5% CO_2_ and 21% O_2_ unless otherwise specified i.e. for hypoxia experiments. Cells tested negative for mycoplasm contamination and their identity was confirmed by STR profiling, where possible, by the CRUK Cambridge institute’s research instrumentation and cell services (RICS) core facility. HUVECs (ATCC, CRL-1730, RRID:CVCL_2959, Female) were grown in EBM-2 Endothelial Cell Growth Medium-2 Bullet Kit (Lonza, CC-3156) at 37°C with 5% CO_2_ and 21% O_2_.

## Method details

### Virus production and cloning

For VSVG-pseudotyped virus production, Platinum-A retroviral packaging cells (Cell Biolabs Inc, RV-102) were transfected using the CalPhos mammalian transfection kit (Clontech, 631312). An MSCV-based retroviral vector harboring a bi-cistronic transcript (mCHERRY-IRES-Hygromycin) downstream of the PGK promoter, UM-mCherry-Hygro, was used for most shRNA knockdown experiments^37^. For Fig 3F, S3C, S3D, S3G, shRNAs targeting human FOXC2 were cloned into a lentiviral vector containing akaLuciferase and tdTomato using Gibson assembly. To construct the tdTomato-Akaluc vector, tdTomato and p2A-Akaluc were amplified by PCR and cloned into the 3^rd^ generation lentiviral pZIP backbone harbouring a spleen focus-forming virus promoter (SFFV) using Gibson Assembly. Lentivirus production was achieved by co-transfecting 293 FT cells with the transfer plasmid (32 μg) and standard third-generation packaging vectors pMDL (12.5 μg), CMV-Rev (6.25 μg) and VSV-G (9 μg) using the calcium-phosphate transfection method (Clontech, 631312). The transfection mixture was added to the packaging cells along with 100 mM chloroquine (Sigma-Aldrich). After 16–18 hr, media was replaced for fresh growth media. Viral supernatant was collected 48 hr after transfection and filtered through a 45 μm filter. The viral supernatant was applied directly to cells or stored at 4 °C for short-term storage. For acLDL assays in Fig 3F and S3G cells were not selected following transduction so as to have a mixed culture of tdTomato positive and negative cells with non-transduced cells used as internal negative controls. The CellTag vector (pSMAL-CellTag-V1) was purchased from addgene (#115643), transduced cells were sorted on GFP. For cDNA over-expression, the coding sequence of murine Foxc2, fused to a C-terminal FLAG tag, was cloned into pLHCX (Clontech, 631511). For all experiments, infected cells were selected with 400 ug/mL Hygromycin (Thermo Fisher, 10687010) for 48 hrs then recovered in medium lacking antibiotic for at least two days. For inducible shRNA experiments Foxc2 shRNAs were cloned into the TRMP-VIR vector and sorted for zsGreen (constitutively expressed) and expanded. Induction of shRNAs was achieved in cell culture by adding of 5 ug/mL Doxyclyline to the medium for 96 hrs prior to the experimental end point. Induction of shRNAs *in vivo* was achieved by administration of Doxycycline containing food, for details see below.

### Mouse tumor implantation, drug treatments and tomato lectin administration

To assess VM vessel perfusion status, BALB/c mice were implanted with 50,000 unmodified or zsGreen-labelled 4T1-T cells as outlined above. At day 16-18 post implantation, animals were anaesthetized with isoflourane and injected intravenously with 200 μl of 1mg/mL Lycopersicon Esculentum (Tomato) Lectin (LEL, TL), DyLight 649 (Vector Labs, DL-1178-1) followed 15 minutes later by intra-cardiac perfusion with 4% paraformaldehyde (PFA) in PBS. Tumors were excised and post-fixed in 4% PFA overnight at 4 °C with rotation. Following fixation, tumors were washed three times for two hours each in PBS. Tumors were cut into 1 mm thick slices and transferred to 50% CUBIC-L (TCI Chemicals, T3740) for at least 6 hrs at 37 °C with gentle agitation. Tumors were then transferred to 100% CUBIC-L at 37 °C with gentle agitation for 5 days with replacement with fresh of CUBIC-L every 2 days. Samples were again washed three times for two hours each in PBS then incubated in primary anti-CD31/Pecam1 antibody (Cell Signaling Technologies, D8V9E, #77699) diluted 1 in 100 in PBS for 4 days with mixing. Following primary antibody incubation, samples were washed three times in PBS containing 0.1% Triton X100 for 2hrs each at room temperature. Samples were incubated with secondary antibody (Thermo Fisher Scientific, goat anti-rabbit AlexaFluor555) diluted 1 in 1000 in PBS for 4 days with mixing. Following secondary antibody incubation, samples were washed three times in PBS containing 0.1% Triton X100 for 2hrs each at room temperature. Tumors were then incubated with 50% CUBIC-R+ (TCI Chemicals, T3741) for > 6hrs at room temperature followed by overnight incubation in 100% CUBIC-R+ and storage prior to imaging.

### Lightsheet microscopy, image processing and quantification

Imaging was performed on the Zeiss Lightsheet Z.1 system (Carl Zeiss, Germany), equipped with two sCMOS PCO edge cameras (1920 × 1920 pixels, 16-bit), using a 5x dry detection objective lens (EC Plan-NEOFLUAR 5x/0.16 ZEISS), with 1.3 zoom. Samples were attached to a dedicated custom-made holder, and submerged into the imaging chamber filled with ~20ml of the CUBIC-R+ solution, positioned directly between the light-sheet illumination objectives (5× /0.1 ZEISS). Z-stack imaging (3.25 mm/4.7μm z-step size) was preformed using dual side illumination (left and right) and tile scanning (7x8 tiles, with 10% overlap). Pivot scan mode was used in order to reduce shadows artefacts. Two imaging tracks were used with 561 nm and 638 nm excitation lasers for CD31-AF555 and Lectin-DyLight 649 respectively, both with 80 ms exposure time. The laser was blocked with the LBF 405/488/561/640 filter, and the beam was split by an SBS LP 640 splitter. Channel 1 was recorded with BP 575–615, and Channel 2 with LP 660 filters. Individual tiles acquired by the dual side illumination mode, were fused using Zen software, and then stitched to reconstruct the full view image using arivis Vision 4D software, which was used also to create maximum intensity projections MIP (Z-project), and to export selected figures. Images were processed and analysed using arivis Vision 4D software. A representative ROI (1.71x1.78mm), capturing the 3 different regions of the tumour (Region 1: periphery, Region 2: healthy tumor, Region 3: peri-necrotic, where necrotic regions appear out of focus) as shown in Figure S1, was defined and 2 subsets, A and B, for each one of these regions were considered. First, a discrete Gaussian denoising filter was applied to the subset images, as a pre-processing step, over the entire acquired volume (691 z-planes) for both channels (CD31 signal in yellow, and Lectin signal in magenta). Then, a segmenter that includes a combination of automatic seed finding based on structural information of an object map, and a watershed algorithm, was performed for each channel individually in order to extract the 3D vessels’ structure. Parameters like average diameter of vessels, probability threshold (which computes an object probability map based on structural and intensity information) to detect the individual objects, and split sensitivity, were used to fine-tune the segmentation operation. Following segmentation, compartments operation was used in order to identify, and group, the double positive vessels. This segments’ operation groups segments primarily based on overlap (fully contained or partially overlapping). For this analysis, we have used a voxel overlap criteria of 10% between the Lectin and CD31 segments, to account for any possible staining artefacts and avoid underestimation of the double positive vessels. Finally, total vascular volume (μm^3^), per segment type, was calculated in order to quantify the VM-only vessels in each one of the 6 subsets, corresponding to the 3 different regions of the tumour. Confocal imaging was performed on an inverted Leica STELLARIS 8 system (Leica, Germany), equipped with a white light laser, using a 10x dry objective lens (HC PL APO CS 10x/0.40 DRY Leica). The sample was positioned directly into a glass-bottom petri dish filled with the CUBIC-R+ solution. Z-stack imaging (103 steps/12mm z-step size) was preformed combined with tile scanning (9x4 tiles, 10% overlap). The pinhole was set to 1 Airy Units. Images were captured sequentially, with: 488nm, 553 nm and 638 nm excitation lasers for zsGreen, CD31-AF555 and Lectin-DyLight 649 respectively. Channel 1 was recorded on a hybrid detector (Leica Power HyD S) 498-554nm, Channel 2 on a hybrid detector (Leica Power HyD S) 563-638nm, and Channel 3 on a hybrid detector (Leica Power HyD X) 648-737nm. Individual tiles were stitched using LAS X software (Leica) to reconstruct the full view image (4.30x2.04x1.22mm). Deconvolution was performed using the LAS X Lightning package (Leica).

### Bulk gene expression analysis

Gene expression data for FOXC2 over expression in HMLER cells was previously described (GSE44335)^11^, log_2_ fold change values and FDR-corrected p values were generated using the Limma package in R using default settings. RNA-Seq data from MDA-MB-231 cells with FOXC2 knockdown and acLDL uptake RNA-Seq of 4T1-T^VM^ was generated for this study. RNA sequencing libraries were prepared using the TruSeq Stranded mRNA Library Preparation Kit (Illumina) in accordance with the manufacturer’s instructions. Briefly, 500 ng of total RNA was used for purification and fragmentation of the mRNA. Purified mRNA underwent first and second strand cDNA synthesis. cDNA was then adenylated, ligated to illumina sequencing adapters, and amplified by PCR (using 8 cycles). Final libraries were evaluated using fluorescent-based assays and were sequenced on an Illumina HiSeq 2500 (v4 chemistry) using 2 x 50 bp cycles. Raw reads were aligned to hg19 or mm10 using STAR^38^ and gene quantification was performed using featureCounts from the subreads package. RNA-Seq data for all 23 4T1-derived clones has been described previously (GSE63180). Differential expression analysis, log_2_ fold change estimation, and FDR p-value calculation were done with DESeq2^39^ (version 1.20.0) with analysis restricted to protein coding genes. Counts generated using featureCounts were used to determine expression differences of both FOXC2 hairpins relative to a control Renilla hairpin or between clones 4T1-E^VM^ and 4T1-T^VM^ and all the other 4T1 clones or between acLDL high and low populations. Replicate and hairpin information was included in the comparison. betaPrior was set to TRUE resulting in log_2_ fold change shrinkage useful for ranking of genes. Gene set enrichment analysis was performed using ranked lists based on the log_2_ fold change (generated by Limma for microarray data or by DESeq2 for RNA-Seq data) and human gene symbols. Where necessary, mouse gene symbols were converted to human using biomart annotations. These log_2_ fold change ranked lists were used as inputs to the pre-ranked feature of the GSEA java application. Gene sets were either literature curated or from the molecular signatures database (mSigDB) from the “C2_All_V7.2” collection of molecular and cellular perturbations. Full custom gene sets used for GSEA and other analyses are listed in Table S1. Contained within that file are the original version of the Butler et al., list of endothelial enriched genes and two refined versions; one that was used for GSEA and has any mesenchymal genes from Evrard et al., (Endo Gene Set #1, Table S1) removed; and a second refined list that was filtered based on our scRNA-Seq data and used for AUCell analysis of the single cell data. For that refinement, we analyzed expression of the genes in the original Butler et al., list in endothelial cells and fibroblasts from parental 4T1 tumors treated with vehicle, genes that showed greater than 0.5 log_2_ fold change between endothelial cells and fibroblasts (i.e. higher in endothelial cells) and less than 30% of cells within the fibroblast cluster expressing them were included (Endo Gene Set #2). Also within Table S1 are three FOXC2-target gene sets: **(1)** The top 100 up-regulated genes in HMLER cells with FOXC2 over-expression (FOXC2-target Gene Set #1) **(2)** a second more stringent set of FOXC2-target genes, which are genes that are significantly up-regulated with FOXC2 over-expression in HMLER cells and also significantly down-regulated in MDA-MB-231 cells with FOXC2 knockdown (FOXC2-target Gene Set #2) and **(3)** our 5 core FOXC2-target genes defined as follows; we overlaid genes that were significantly up-regulated in HMLER cells with FOXC2 over-expression and significantly down-regulated with FOXC2 knockdown in MDA-MB-231 cells and then required that they show a log_2_ fold change >0.5 in the following comparisons: lung_vs_primary (mouse 4T1) and VM_vs_All (CCLE human) and were highly significantly (FDR < 1 x 10^-5^) up-regulated in VM 4T1 clones vs all other clones. We then ranked genes meeting those criteria by their log_2_ fold change in the 4T1 (VM vs all other clones) comparison and took the top 5 as our high confidence VM FOXC2-target Gene Set #3. For some figures the log10 transformation of the FDR q value derived from GSEA is signed according to whether the enrichment score was positive or negative, with positive values indicating enrichment in up-regulated genes and negative values indicating enrichment in down-regulated genes. Gene expression data from Cancer Cell Line Encyclopedia (CCLE) data were downloaded from cBioPortal (http://www.cbioportal.org) and were re-Z-scored having removed hematopoietic cell lines from the analysis. Breast cancer cell line intrinsic subtypes were curated from a combination of Prat et al., and Heiser et al.,^40,41^. We curated from the literature a high confidence set of human cell lines that convincingly form tube structures in Matrigel HCC38 (Fig S2A), MDA-MB-231 (Fig 2A and S2A), U87MG^21^, NCIH446^18^, U251MG^42^ in ours or other investigators experiments. To derive a ranked list for GSEA analysis, the median of each gene across the 5 VM-proficient cell lines was calculated and the difference between that median and the global median for all cell lines formed the ranking metric for GSEA. CDX RNA-Seq data is described in Pearsall et al.,^13^

### CCLE proteomics analysis

Quantile normalized protein expression measurements were downloaded from the supplementary data of Nusinow et al.,^43^. For GSEA analysis the mean Z-score of each protein was calculated across the VM competent cell lines and the rest of the solid tumor cell lines in the dataset and the difference between the means was used as a ranking metric for the pre-ranked function of GSEA.

### Single cell RNA-Seq

4T1-T^VM^ or 4T1 CellTag-labelled^29^ parental tumors +/- Axitinib treatment were dissociated using Miltenyi tumor dissociation kit (mouse) (#130-096-730) and the gentleMACS Octo Dissociator with heaters from Miltneyi Biotec as described previously^44^ according to the supplied protocol. Briefly, Tough tumor dissociation protocol was used the program 37_m_TDK_2 followed by program m_impTumor_01 the resulting cell suspensions were submitted for single-cell RNA-seq library preparation at the Cancer Research UK Cambridge Institute Genomics Core Facility using the following: Chromium Single Cell 3′ Library & Gel Bead Kit v3, Chromium Chip B Kit and Chromium Single Cell 3’ Reagent Kits v3 User Guide (Manual Part CG000183 Rev A; 10X Genomics). Cell suspensions were loaded on the Chromium instrument with the expectation of collecting gel-beads emulsions containing single cells. RNA from the barcoded cells for each sample was subsequently reverse-transcribed in a C1000 Touch Thermal cycler (Bio-Rad) and all subsequent steps to generate single-cell libraries were performed according to the manufacturer’s protocol with no modifications (12 cycles was used for cDNA amplification). cDNA quality and quantity was measured with Agilent TapeStation 4200 (High Sensitivity 5000 ScreenTape), after which 25% of material was used for gene expression library preparation. Library quality was confirmed with Agilent TapeStation 4200 (High Sensitivity D1000 ScreenTape to evaluate library sizes) and Qubit 4.0 Flourometer (Thermo Fisher Qubit™ dsDNA HS Assay Kit to evaluate dsDNA quantity). Each sample was normalized and pooled in equal molar concentration. To confirm concentration pools were qPCRed using KAPA Library Quantification Kit on QuantStudio 6 Flex before sequencing. Pools were sequenced on Illumina NovaSeq6000 sequencer with following parameters: 28 bp, read 1; 8 bp, i7 index; and 91 bp, read 2. The Cell Ranger v3.0.1pipeline (https://support.10xgenomics.com/single-cell-gene-expression/software/down-loads/latest) was used to process data generated by the 10X Chromium platform. The pipeline relies on STAR for alignment and was used in conjunction with a custom reference genome, created by adding the sequence of the Celltag-V1 barcode transgene as a new chromosome to the mm10 mouse genome. To create a new reference genome compatible with Cell Ranger, we followed the instructions from 10XGenomics on building a custom reference (https://support.10xgenomics.com/single-cell-gene-expression/software/pipelines/latest/using/tutorial_mr#runmkref). In brief, we first modified the reference genome used during alignment by adding the full lentiviral plasmid sequence including the transgene above. We then created a custom gene transfer format (GTF) file, containing our custom transgene annotation, followed by indexing of the FASTA and GTF files, using the Cell Ranger mkref function.

Single cell RNA-seq analysis was performed using Seurat 3.0 and a standard analysis pipeline. Briefly, features were excluded that were not detected in at least 5 cells per sample. Cells were filtered for unique feature counts in the range 200 to 9000 and less than 10 percent of reads mapping to the mitochondrial genome. SCTransform normalisation was applied to the data including regression of cell cycle phase scores. The two replicates of 4T1-T^VM^ tumor datasets were further analysed as a combined dataset without integration due to high levels of reproducibility between replicates. To facilitate assignment of cells to clusters with and without treatment, the datasets from two vehicle treated mice and two Axitinib treated mice were integrated using a reciprocal PCA strategy as described in Stuart et al.,^45^ with the number of integration features set to 5000 and using one vehicle sample and one Axitinib sample as the reference. Clustering of the integrated Axitinib dataset gave 22 separate cell clusters. Analysis of the expression of the CellTag transcript as a marker of tumor cells or Pecam1 and Cdh5 as markers of endothelial cells across the clusters identified three clusters containing tumor cells and one cluster of endothelial cells. The endothelial cell cluster was subsetted and pseudo-bulk analysis performed using DESeq2^39^ (version 1.20.0). Briefly, the counts matrix for cells belonging to this cluster were aggregated by sample to give the sum of counts per gene per sample and DESeq2 was used to compare the two vehicle samples with the two Axitinib treated samples with log fold change shrinkage type set to ‘normal’.

For the 4T1-T^VM^ dataset, the tumor cell clusters were subsetted and the expression of gene signatures across the tumor cells was analysed using AUCell^46^. AUCell uses a rank-based scoring method applied to each cell individually that enables the relative expression of gene sets across all cells in a scRNA-seq dataset to be explored. AUCell was run with default setting with the exception of the aucMaxRank parameter which was set to 10% of the total number of genes in the ranking. To define the endo-high population in 4T1-T tumors, we calculated AUCell scores across the tumor cells using Endo Gene Set #2. We then compared the top 5% of cells based on this score to the rest of tumor cells using the find markers function in Seurat. The log_2_ fold change values from this analysis were used as the ranking metric for further GSEA analysis. A similar procedure was performed for Axitinib treated tumors. Since the vehicle tumors had lower endo scores, as expected, we restricted our analysis to the top 5% of cells within the Axitinib treated tumors. Comparison of the percentage of cells meeting the numerical AUCell value cut-off in are illustrated in Fig 5G for vehicle and Axitinib tumors and show less than 2% of the tumor cells in the vehicle tumors are endo-high by this metric.

### VM network formation assay

24 hrs prior to reseeding for tube assays 4T1, 4T1-T, MDA-MB-231, HCC38 or HUVEC cells were seeded at a density of 400,000 cells/10cm dish in complete growth medium. 24 well plates were pre-coated with ice cold growth factor-reduced Matrigel (Corning, protein content >9, 356231) and then allowed to set at 37 °C for at least 2 hrs. 4T1-Ts were trypsinized, counted and put through a 40 μm cell strainer. Cells were then seeded onto the pre-coated Matrigel plates at 120,000 cells per well in EBM-2 Basal medium (Lonza, CC-3156) lacking any additives and imaged by microscopy 16 hrs later on an Invitrogen EVOS FL microscope. 3-5 images were taken per well and total branching length determined by automated analysis using the angiogenesis analyzer plug-in for imageJ. The average total branching length from the 3-5 images was taken as one replicate and each experiment was repeated on at least 3 separate days, as indicated in the figure legends. For CDX17 cultures, SCLC cells were seeded onto 6 well plates at a density of 1.5 x 10^6^ cells and imaged by phase contrast microscopy after 24 hours.

### CDX Generation

CDX models were generated as previously described^47^. In brief, 10mL of EDTA peripheral blood was collected from SCLC patients enrolled onto the CHEMORES study (07/H1014/96). CTCs were enriched via RosetteSep™ (#15167, Stem Cell Technologies) and subcutaneously implanted into the flank of 8-16 week old non-obese diabetic (NOD) severe combined immunodeficient (SCID) interleukin-2 receptor γ–deficient (NSG) mice (Charles River). CDX models were generated from patients CTCs enriched from blood samples at pre-chemotherapy baseline and/or at post-treatment disease progression time-points (designated P, or PP). All procedures were carried out in accordance with Home Office Regulations (UK), the UK Coordinating Committee on Cancer Research guidelines and by approved protocols (Home Office Project license 40-3306/70-8252 and Cancer Research UK Manchester Institute Animal Welfare and Ethical Review Advisory Board).

### Disaggregation and culture of CDX

CDX tumors were removed at approximately 800mm^3^ and dissociated into single cells using Miltenyi Biotecs tumor dissociation kit (#130-095-929) following the manufacturer’s instructions on a gentleMACS octo dissociator (#130-096-427), as previously described^47,48^. Single cells were incubated with anti-mouse anti-MHC1 antibody (Thermo Fisher Scientific eBioscience, clone #34-1-2s), anti-mouse anti-igG2a+b microbeads and Dead cell removal microbead set (Miltenyi Biotecs, 130-090-101) and applied to an LS column in a MidiMACS Seperator for immunomagnetic depletion of mouse cells and dead cells. CDX *ex vivo* cultures were maintained in RPMI supplemented with HITES components (10nM Hydrocortisone, 0.005 mg/mL Insulin, 0.01 mg/mL Transferrin, 10 nM β-estradiol, 30nM Sodium selenite), Rock inhibitor added fresh (Selleckchem, Y27632), and 2.5% FBS added after one week at 37 °C and 5% CO_2_.

### Acetylated low-density lipoprotein (acLDL) uptake assay

4T1-derived cells were seeded at 40% confluency in 6 well plates. 24 hr later they were swapped to serum starvation medium, consisting of Opti-Mem (Invitrogen) supplemented with 0.3% BSA, and incubated overnight. The next morning Alexa-488 labelled ac-LDL (Thermo Fisher, L23380) was added at a final concentration of 10 ug/mL and cells were incubated for 4 hours. Cells were washed 8 times in PBS (+Ca and Mg), then further incubated in complete growth medium for 1 hour and imaged. Positive cells per field were counted using the threshold function in imageJ. The average number of positive cells over 3-5 images per condition was taken as one replicate and each experiment was repeated on at least 3 separate days, as indicated in the figure legends. For U86-MG and H446 acLDL uptake assays, 6-well cell culture plates were first coated with Collagen coating solution (Sigma-aldrich, 125-50) for 2 hrs at 37°C followed by aspiration of excess solution prior to seeding. For 4T1-T^VM^ FACS sorting subsequent to acLDL incubation, cells were trypsinized and resuspended in MACS buffer (1x PBS, 0.5% BSA, 2 mM EDTA) and put through a 40 μm cell strainer. Cells were then sorted using a Sony SH800 cell sorter using the EGFP gate taking the top 25% as high and bottom 25% as low uptake these gates were also used for samples collected for RNA-Seq analysis of which is described above.

### RNA extraction and qRT-PCR

RNA was extracted using either Trizol or the High Pure RNA isolation kit (Roche, 11828665001) according to the manufacturers’ instructions. For qRT-PCR, 1-5 μg of total RNA was reverse transcribed using oligodT and random hexamers and the Superscript III reverse transcription kit (Invitrogen). Resultant cDNA was used as a template for qPCR with gene-specific primers and the SYBR green master mix (Applied Biosystems, 4309155) for 40 cycles on a Bio-Rad C1000 Thermal Cycler. Data were analyzed using the delta-delta Ct method as described previously^49^ using Ubc/UBC as a housekeeping gene.

### Cell viability assays and Hypoxia induction

Cell viability was measured using CellTiterGlo 2.0 (Promega, G9242). Cells were seeded at density of 10,000 cells per well in a 96 well plate (8 technical replicates per condition). The next day media was aspirated and 100 μl of 1:1 CellTiterGlo:PBS was added, the plate was placed on shaker for 5 minutes, then incubated at room temperature for 10 min and bioluminescence was measured in a Tecan Infinite 200 Pro plate reader. To generate hypoxia exposed cells, 4T1 parental cells or those with Foxc2 knockdown were seeded at density of 1,000,000 cells/10 cm dish in duplicate. The next day, one plate was placed in avatar incubator at 0.1% O_2_ and the other in a standard tissue culture incubator at atmospheric Oxygen levels for 24 hrs. Cells were recovered for 24 hrs in a standard tissue culture incubator at atmospheric Oxygen levels prior to replating for matri-gel tube assays. For hypoxia viability assays, cells were plated in 96 well plates as above and subjected to 0.1% O_2_ in an avatar incubator for 72hrs or maintained at atmospheric oxygen under standard tissue culture conditions, then immediately processed for CellTiterGlo assays.

### CD31/PAS staining

Detection of mouse CD31 (Cell Signalling, 77699) was performed on 3μm thick, re-hydrated FFPE sections using Leica’s Polymer Refine Detection System (Leica Biosystems, DS9390), and a modified version of their standard template on the automated Bond RX. Briefly, antigen was retrieved using Epitope Retrieval Solution 2 (Leica Biosystems, AR9640) at 100 °C for 20 minutes. The CD31 antibody was diluted 1:100 in Bond Primary Antibody Diluent (Leica Biosystems, AR9352) and detected with anti-rabbit poly HRP-IgG. Signal was intensified using Bond DAB Enhancer (Leica Biosystems, AR9432). Slides were removed from the Bond RX, washed well in ultrapure water and incubated for 5 minutes in fresh 0.5% periodic acid (Sigma-Aldrich, P0430). Following further washing in ultrapure water, sections were incubated for 30 minutes in Schiff’s reagent (Thermo Fisher, J/7300/PB08) for the detection of aldehydes. Washed sections were dehydrated, cleared in xylene on the Leica ST5020 (Leica Biosystems) and mounted in DPX on the CV5030 (Leica Biosystems). The slides were imaged at 20x magnification on the Aperio AT2 (Leica Biosystems), with a resolution of 0.5 microns per pixel. Two 20x images were then taken from each tumor from the central mass of the tumor (2 individual tumors from individual mice per condition), in the same regions across tumors. Images were manually quantified, in a blinded fashion, by counting PAS positive channels with vessel-like morphology that were negative for CD31 expression.

### Analysis of clinical data

For METABRIC analysis gene expression data and subtype information were downloaded from cBioPortal (http://www.cbioportal.org). For gene signatures, a mean Z score was calculated across all genes for that signature, per patient, and then all patients belonging to a given subtype were plotted as a group. For Fig S4K, a normalized gene expression matrix was kindly provided by Oscar Rueda. For colorectal cancer data were from originally reported by Marisa et al.,^50^ and gene expression and subtype calls were downloaded from the Colorectal Cancer Subtyping Consortium’s^51^ synapse page (www.synapse.org) under synapse ID syn2634724. All other patient data for other tumor types was from TCGA and was gene expression data downloaded from the TCGA GDAC Firehorse (https://gdac.broadinstitute.org/) and clinical annotations were downloaded from cBioPortal (http://www.cbioportal.org). In all cases, gene expression for the 5 core FOXC2-target genes was extracted and these values were Z-scored across patients and then mean Z-score of the 5 core FOXC2-target genes was calculated per patient. The mean Z-score of the 5 core FOXC2-target genes was then used as a variable against which to correlate all other genes in the dataset. Correlation coefficients from these analyses were used as a ranking metric for GSEA analogous to the approach used by VISAGE ^52^. In all figures presenting patient data, n represents an individual patient. For analysis of the ICON7 trial gene expression data was downloaded from the gene expression omnibus (GEO) under accession GSE140082^53^ and analysed as described above for other datasets.

### Patient-derived tumor xenograft (PDTX) analysis

The species mapping approach has been described^28^ and the data are available through the European Genome-Phenome Archive (EGA, https://ega-archive.org/) under accession number EGAD00001003800 and the gene expression data from all models has been described^27^ and is available under the accession number EGAS00001001913. Briefly, we curated a non-redundant set of samples representing different PDTX models. Primary tumors and metastases from the same patient were treated as separate entities. Where data from multiple passages of PDTXs were available the later passage was taken under the assumption it was less likely to contain human endothelial cells from the original tumor that would confound our analysis. FOXC2-target gene expression was calculated as outlined above. FOXC2-target gene expression was correlated with all other genes in the human dataset or mouse dataset and the correlation coefficients from this analysis used as the ranking metric for GSEA.

### FOXC2 immunofluorescence of PDTX tissue

3μm FFPE PDTX sections were dewaxed in the following solutions: 2x xylene 5 min, 100% ethanol 20 s, 90% ethanol 20 s, 70% ethanol 20 s, 2x water 20 s. Antigen retrieval was performed at 100 °C in 10mM tri-sodium citrate, pH 6.0 for 10 minutes followed by a brief wash in PBS. The tissue was permeabilised using 0.2% Triton X-100 in PBS for 30 minutes, followed by three PBS washes for 5 mins with gentle rocking. Tissue was blocked in blocking buffer (10% Goat Serum, 150mM Maleimide, 100mM Ammonium Chloride in PBS) for one hour at room temperature. Anti-human FOXC2 (clone 1D4, Novus Biologicals, H00002303-M16) was diluted to 10μg/ml in staining buffer (5% Goat Serum, 100mM Ammonium Chloride in PBS) and incubated on the slides overnight at 4 °C within a humidity chamber. Slides were washed three times in PBS for five minutes. DAPI (BD Biosciences, 564907) and goat anti-mouse IgG Alexa Fluor 488 (ThermoFisher Scientific, A-11001) were diluted to concentrations of 1μg/ml and 7μg/ml respectively in staining buffer and incubated on the slides at room temperature for two hours. Slides were washed three times in PBS for five minutes with gentle rocking. Imaging buffer (700mM N-Acetyl-cysteine in water, pH 7.4) was added and the tissue was imaged at 20x using the Leica DMI 4000b system using the same acquisition settings for each slide. Images were analysed on Indica Labs Halo® Image Analysis Platform using the CytoNuclear FL v2.0.12 algorithm. Briefly, tissue edges and necrotic areas were manually excluded from analysis and the remaining nuclei were segmented using the DAPI stain. Each sample contained between 10,000-60,000 cells. FOXC2 fluorescence intensities were categorised into weak, moderate and strong for each nucleus to calculate the H-score.

### shRNA cloning and sequences

The shRNA sequences (see supplementary table S4) were purchased as 97mer oligonucleotides from Invitrogen and used as a template for a PCR with the below primers and cloned into UM-mCherry-Hygro by Gibson assembly: HpHpaI Fwd: CTGGGATTACTTCTTCAGGTTAACCCAACAGAAGGCTAAAGAAGGTATATTGCTGTTGA CAGTGAGCG HpHpaI Rev: AGAGATAGCAAGGTATTCAGTTTTAGTAAACAAGATAATTGCTCCTAAAGTAGCCCCTTG AAGTCCGAGGCAGTAGGCA

For TMRP-VIR cloning the same shRNA sequences were amplified by PCR with the below primers and cloned into TMRP-VIR by Gibson assembly:

XhoI Fwd: CAGAAGGCTCGAGAAGGTATATTGCTGTTGACAGTGAGCG EcoRI Rev: CTAAAGTAGCCCCTTGAATTCCGAGGCAGTAGGCA

The sequences listed in table S4 are the templates used for PCR. In bold are the gene-specific unique regions of the hairpin.

### qRT-PCR primer sequences

Mmu Foxc2 qPCR Fwd: ACAGTTGGGCAAGACGAAAC Mmu Foxc2 qPCR Rev: AGTGCGGATTTGTAACCAGG Mmu Ubc qPCR Fwd: GACGTCCAAGGTGATGGTCT Mmu Ubc qPCR Rev: TCCAGAAAGAGTCCACCCTG Hsa FOXC2 qPCR Fwd: AGTTCATCATGGACCGCTTC Hsa FOXC2 qPCR Rev: TCTCCTTGGACACGTCCTTC Hsa UBC qPCR Fwd: ATCGCTGTGATCGTCACTTG Hsa UBC qPCR Rev: TTGCCTTGAC

### Quantification and statistical analysis

Details of statistical tests used and definitions of n can be found in the figure legend to each figure. Unless otherwise stated student t-tests and Wilcoxon rank-sum tests were two tailed and were calculated in GraphPad Prism or Seurat (for single cell analyses). The Wilcoxon rank-sum test was used for patient sample analyses and other unpaired groups with n >7 as it has little power below this. Students t-tests were used when the data could be assumed to be randomly sampled, with homogenous variation and an approximately normally distribution. No specific methods were used to predetermine whether data met the assumptions of a specific statistical test, instead we looked at similar pre-existing data to make these determinations. In all cases a p-value of less than 0.05 was taken as significant.

## Key Resource Table

**Table:** 

REAGENT or RESOURCE	SOURCE	IDENTIFIER
Antibodies		
anti-CD31/Pecam1 antibody D8V9E	Cell Signaling Technologies	Cell Signaling Technology Cat# 77699, RRID:AB_2722705
Goat anti- Rabbit IgG (H+L) Highly Cross-Adsorbed Secondary Antibody, Alexa Fluor™ Plus 555	Thermo Fisher Scientific	Thermo Fisher Scientific Cat# A32732, RRID:AB_2633281
anti-MHC1 antibody	Thermo Fisher Scientific/eBioscience	Thermo Fisher Scientific Cat# 16-5998-025, RRID:AB_2866179
anti-FOXC2 monoclonal antibody (clone #1d4)	Novus Biologicals	Novus Cat# H00002303-M16, RRID:AB_921290
Goat anti-Mouse IgG (H+L) Cross-Adsorbed Secondary Antibody, Alexa Fluor™ 488	Thermo Fisher Scientific	Thermo Fisher Scientific Cat# A-11001, RRID:AB_2534069
Biological Samples		
PDTX FFPE blocks	Carlos Caldas Lab	
Chemicals, Peptides, and Recombinant Proteins		
Axitnib	MedChem Express	Cat# HY-10065
B20-4.1.1	Gift from Genetech	N/A
Doxycycline hyclate	Sigma Aldrich	Cat# D9891
Doxyxycline diet	Bioserv	S3888
Critical Commercial Assays		
Chromium Single Cell 3’ Library & Gel Bead Kit v3	10X Genomics	Cat# PN-1000075
Chromium Single Cell B Chip Kit	10X Genomics	Cat# PN-1000074
Tumor Dissociation Kit (mouse)	Miltenyi Biotec	Cat# 130-096-730
SYBR green PCR master mix	Applied Biosystems/ Thermo Fisher Scientific	Cat # 4309155
High pure RNA isolation kit	Roche	Cat # 11828665001
Deposited Data		
Microarray data of FOXC2 over-expression in HMLER cells	Gene Expression Omnibus	GSE44335
CCLE proteomics data	Supplement of Nusinow et al.,	
ICON7 microarray data	Gene Expression Omnibus	GSE140082
Patient-derived tumor xenograft RNA-seq data	European Genome-Phenome Archive	EGAS0000100191 3
Single cell RNA-seq of 4T1-T tumors and 4T1 parental tumors treated with Axitinib	This paper	GSE230643
Bulk RNA-Seq of MDA-MB-231 cells with FOXC2 knockdown and 4T1-T cells sorted based on acLDL uptake	This paper	GSE232214
Experimental Models: Cell Lines		
MDA-MB-231	ATCC	Cat# HTB-26 RRID:CVCL_0062
HCC38	ATCC	Cat# CRL-2314 RRID:CVCL_1267
NCI-H446	ATCC	Cat# HTB-171 RRID:CVCL_1562
786-O	ATCC	Cat# CRL-1932 RRID:CVCL_1051
U-87 MG	ATCC	Cat# HTB-14 RRID:CVCL_0022
4T1 (Parental)	ATCC	Cat# CRL-2539 RRID:CVCL_0125
293 FT	Thermo Fisher Scientific	RRID:CCVCL_691 1
4T1-T, 4T1-E, 4T1-L	Wagenblast et al., Nature. (2015)	
HUVEC	ATCC	Cat# CRL-1730 RRID:CVCL_2959
Platinum-A	Cell Biolabs Inc.	RV-102
Experimental Models: Organisms/Strains		
Mouse: BALB/ C Female 6-8 weeks old	Taconic Biosciences or Charles River	BALB/cAnNTac (Taconic) Strain code 028 (Charles River)
Oligonucleotides		
shRNA sequences	See methods section “shRNA cloning and sequences” and table S4	
qRT-PCR primers	See methods section “qRT-PCR primers”	
Recombinant DNA		
CellTag vector. pSMAL-CellTag-V1	Biddy et al., (2018).	Addgene Cat# 115643
Software and Algorithms		
Cell Ranger	10X Genomics	RRID:SCR_017344
LIMMA	http://bioinf.wehi.edu.au/limma/	RRID:SCR_01094 3
DESeq2	https://bioconductor.org/packages/release/bioc/html/DESeq2.html	RRID:SCR_01568 7
Seurat	https://satijalab.org/seurat/get_started.html	RRID:SCR_016341
Tissue Specific Enrichment Analysis/ TSEA	http://genetics.wustl.edu/jdlab/tsea/	
Gene Set Enrichment Analysis/GSEA	http://www.broadinstitute.org/gsea/	RRID:SCR_003199
AUCell	https://bioconductor.org/packages/AUCell/	RRID:SCR_02132 7
Angiogenesis Analyzer Macro for ImageJ	https://imagej.nih.goV/ij/macros/toolsets/Angiogenesis%20Analyzer.txt	

## Supplementary Material

Supplementary Materials
