# Supplementary material for: FOXC2 promotes vasculogenic mimicry and resistance to anti-angiogenic therapy
Source: Cell Rep. Author manuscript; Available in PMC 2025 Jun 17. (PMC7617767; doi:10.1016/j.celrep.2023.112791)
Supplement: Supplementary Materials [file EMS206336-supplement-Supplementary_Materials.pdf]

Additional files.

Supplementary table S1. List of gene sets used for analysis.
Supplementary table S2. DESeq2 analysis of MDA-MB-231 cells with FOXC2 knockdown showing endothelial genes and other genes of interest with annotations of function.
Supplementary table S3. Full DESeq2 analysis output of MDA-MB-231 cells with FOXC2 knockdown.
Supplementary table S4. List of shRNA template sequences used for cloning.

Supplemental Text and Figures

A

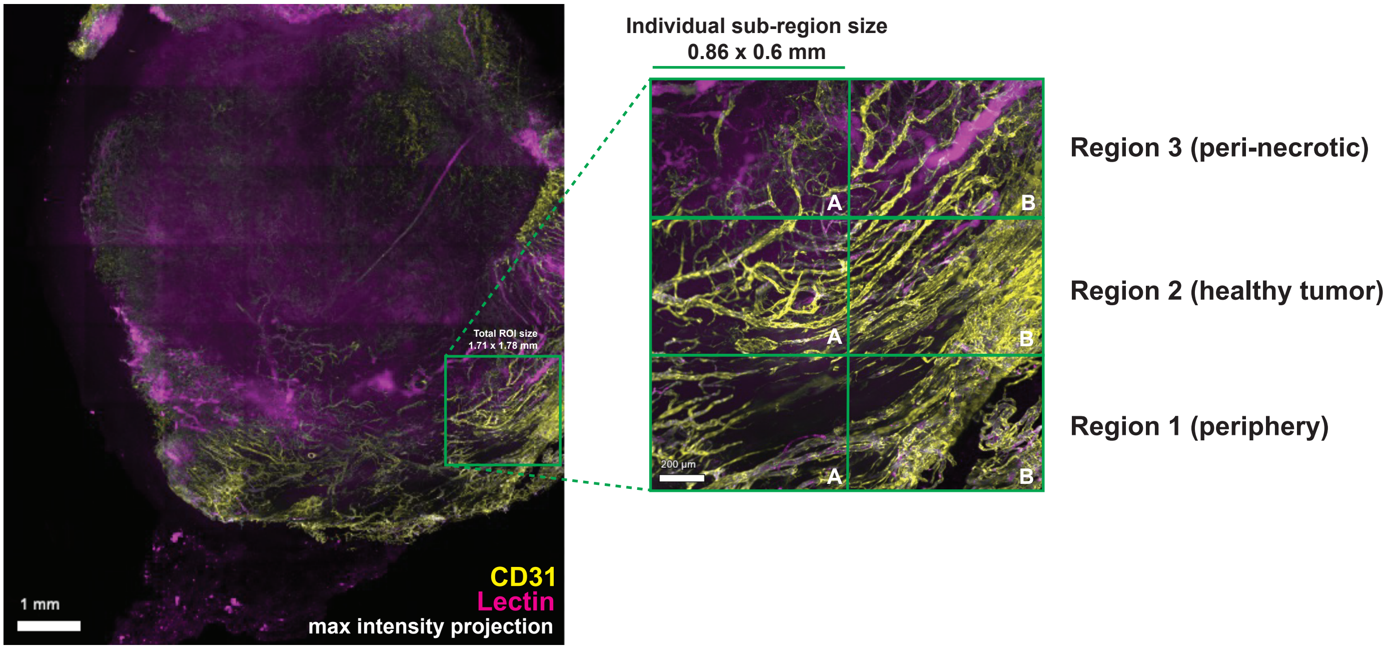

B

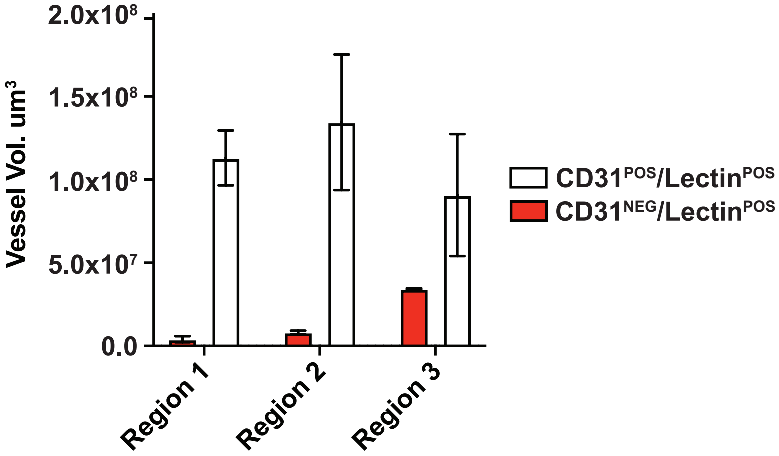

Figure S1. Related to Figure 1. Visualization of perfused vasculogenic mimicry channels *in vivo* with lectin labelling, tissue clearing and 3D imaging.

(A) Maximum intensity projection highlighting the region of interest and the sub-regions used in the quantification analysis.

(B) Output of the quantification analysis showing the total volume of $CD31^{POS}$/$Lectin^{POS}$ (host vessels) and $CD31^{NEG}$/$Lectin^{POS}$ (VM vessels). Bars represent mean volume in μm^3 +/- SEM, n=2 sub-regions per regions.

A

HCC38 Human Claudin-low Breast Cancer

MDA-MB-231 Human Cluadin-low Breast Cancer

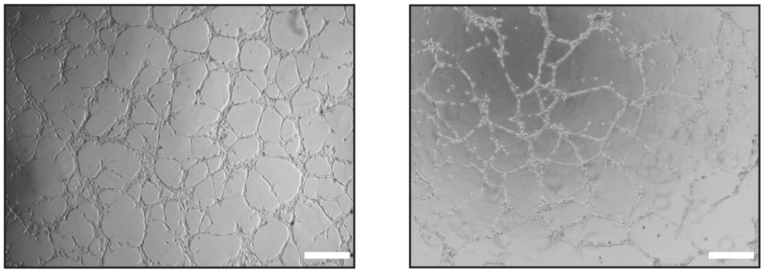

Figure S2. Related to Figure 2. FOXC2 is up-regulated in vasculogenic mimicry-proficient tumor cells.

(A) Exemplar Matrigel network formation assays of human Claudin-low breast cancer cell lines MDA-MB-231 and HCC38. Scale bar = 200 μm.

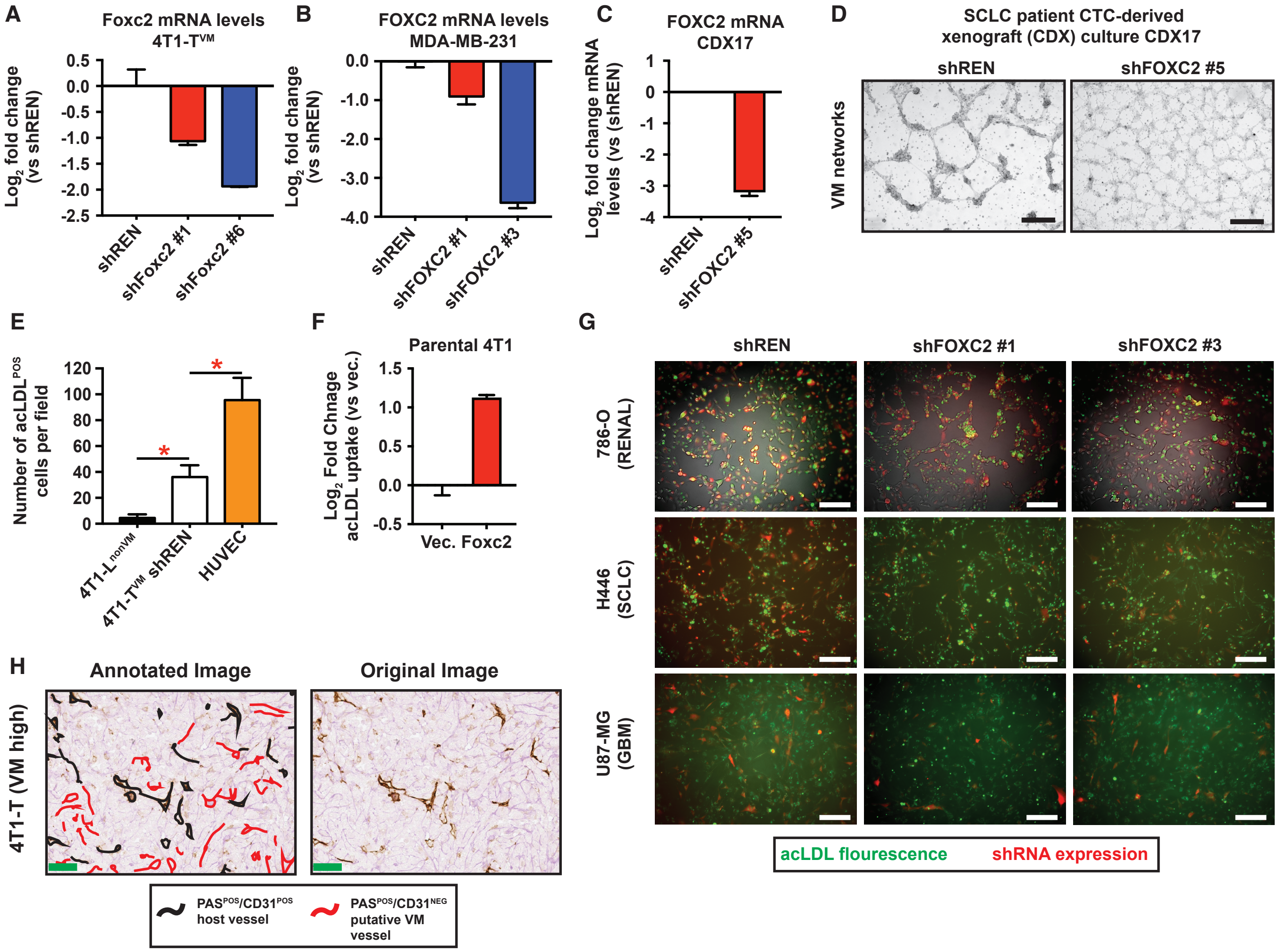

A
Foxc2 mRNA levels
4T1-T^VM
Log2 fold change (vs shREN)
0.5
0.0
-0.5
-1.0
-1.5
-2.0
-2.5
shREN
shFoxc2 #1
shFoxc2 #6
B
FOXC2 mRNA levels
MDA-MB-231
Log2 fold change (vs shREN)
0.0
-1.0
-2.0
-3.0
-4.0
shREN
shFOXC2 #1
shFOXC2 #3
C
FOXC2 mRNA
CDX17
Log2 fold change mRNA levels (vs (shREN)
1
0
-1
-2
-3
-4
shREN
shFOXC2 #5
D
SCLC patient CTC-derived
xenograft (CDX) culture CDX17
shREN
shFOXC2 #5
VM networks
E
Number of acLDL^POS cells per field
120
100
80
60
40
20
0
4T1-L^nonVM
4T1-T^VM shREN
HUVEC
F
Parental 4T1
Log2 Fold Chnage acLDL uptake (vs vec.)
1.5
1.0
0.5
0.0
-0.5
Vec. Foxc2
G
shREN
shFOXC2 #1
shFOXC2 #3
786-O (RENAL)
H446 (SCLC)
U87-MG (GBM)
acLDL flourescence
shRNA expression
H
Annotated Image
Original Image
4T1-T (VM high)
PAS^POS/CD31^POS host vessel
PAS^POS/CD31^NEG putative VM vessel

Figure S3. Related to Figure 3. FOXC2 is required for VM and the endothelial-like properties of VM-proficient tumor cells.

(A) Foxc2 mRNA levels in 4T1-T^{VM} cells expressing two different shRNAs against Foxc2. Bars represent log_2 fold change (+/- SEM) vs control shREN. n = 3 technical replicates.

(B) FOXC2 mRNA levels in MDA-MB-231 cells expressing two different shRNAs against FOXC2. Bars represent log_2 fold change (+/- SEM) vs control shREN. n = 3 technical replicates.

(C) FOXC2 mRNA levels in adherent CDX17[1] cells expressing a control shRNA or an shRNA against FOXC2. Bars represent log_2 fold change (+/- SEM) vs control shREN. n = 2 technical replicates.

(D) Suppression of FOXC2 impedes Matrigel network formation in short term cultures of SCLC CDX cells. Representative matri-gel tube assays with or without FOXC2 knockdown in the adherent cells cultured from CDX17[1]. Scale bar = 500 μm.

(E) Quantification of acLDL uptake in the indicated cell lines. HUVEC are human umbilical vein endothelial cells. Bars represent mean number of acLDL positive cells per field (+/- SEM), n = 3, * $p < 0.05$, student's t-test.

(F) acLDL uptake in parental 4T1s over-expressing FLAG-tagged Foxc2 or empty vector (vec.) assayed by flow cytometry. Bars represent log_2 fold change acLDL positive cells vs vector transduced cells (+/- SEM), n=2 biological replicates.

(G) Representative images of acLDL uptake assays in human cell lines. Green indicates Alexa-488 flourescence i.e. acLDL positive cells, and red indicates tdTomato expression i.e. virally transduced cells. Scale bar = 200 μm.

(H) Annotated example of CD31/PAS staining of 4T1-T tumors. Black lines indicate PAS positive CD31 positive host vessels, Red lines indicate PAS positive CD31 negative putative VM vessels. They are scored according to the following criteria, (1) Vessel-like morphology, (2) PAS positivity, (3) lack of CD31 staining. Any vessels with weak or ambiguous CD31 staining are not quantified. Scale bar = 100 μm.

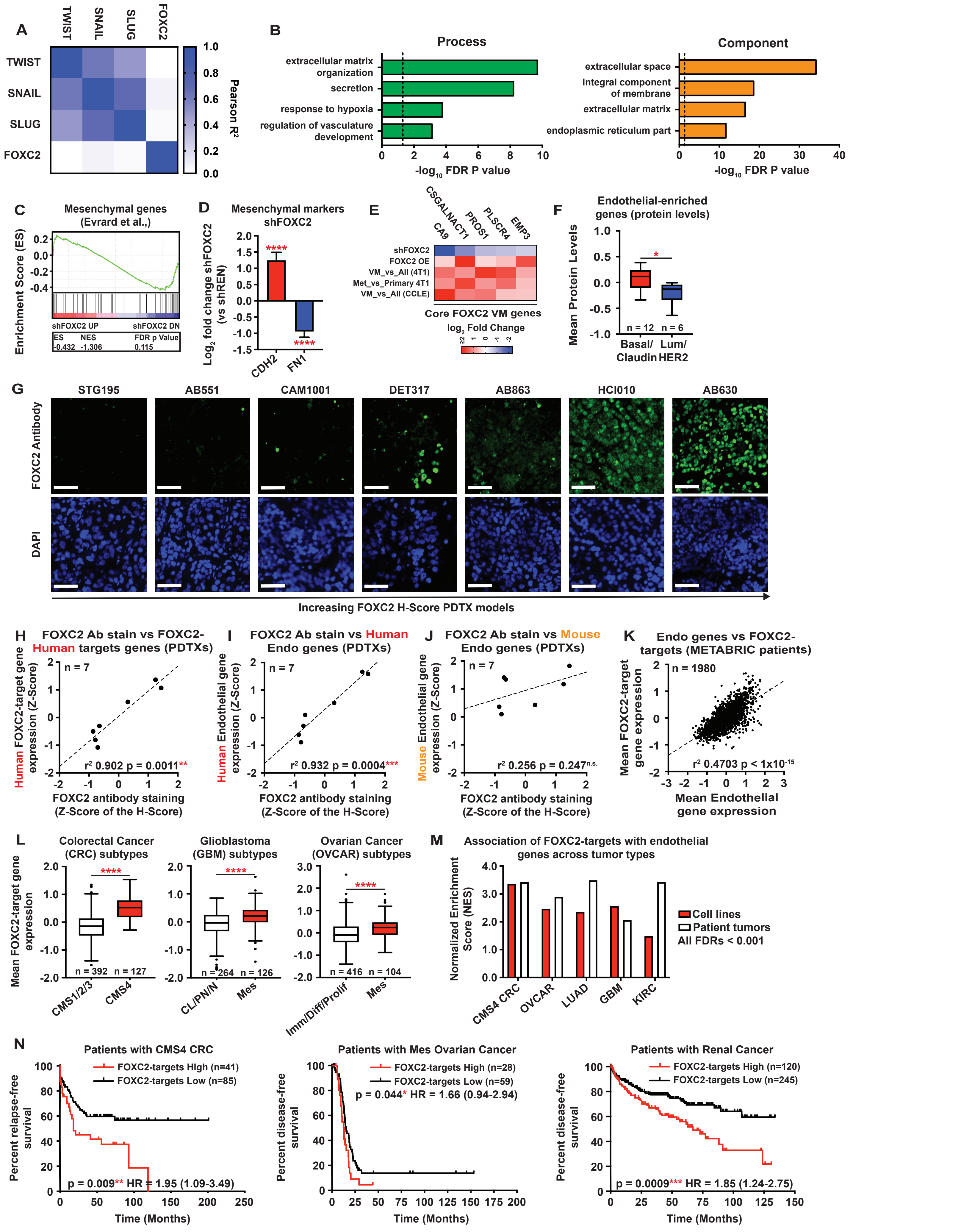
A
TWIST
SNAIL
SLUG
FOXC2
1.0
0.8
0.6
0.4
0.2
0.0
Pearson R²
B
Process
extracellular matrix organization
secretion
response to hypoxia
regulation of vasculature development
-log10 FDR P value
Component
extracellular space
integral component of membrane
extracellular matrix
endoplasmic reticulum part
-log10 FDR P value
C
Mesenchymal genes (Evrard et al.,)
Enrichment Score (ES)
shFOXC2 UP
shFOXC2 DN
ES -0.432
NES -1.306
FDR p Value 0.115
D
Mesenchymal markers shFOXC2
Log2 fold change shFOXC2 (vs shREN)
****
CDH2
FN1
E
CA9
CSGALNACT1
PROS1
PLSCR4
EMP3
shFOXC2
FOXC2 OE
VM_vs_All (4T1)
Met_vs_Primary 4T1
VM_vs_All (CCLE)
Core FOXC2 VM genes
log2 Fold Change
F
Endothelial-enriched genes (protein levels)
Mean Protein Levels
*
n = 12
n = 6
Basal/ Claudin
Lum/ HER2
G
STG195
AB551
CAM1001
DET317
AB863
HCI010
AB630
FOXC2 Antibody
DAPI
Increasing FOXC2 H-Score PDTX models
H
FOXC2 Ab stain vs FOXC2-Human targets genes (PDTXs)
n = 7
r² 0.902 p = 0.0011**
Human FOXC2-target gene expression (Z-Score)
FOXC2 antibody staining (Z-Score of the H-Score)
I
FOXC2 Ab stain vs Human Endo genes (PDTXs)
n = 7
r² 0.932 p = 0.0004***
Human Endothelial gene expression (Z-Score)
FOXC2 antibody staining (Z-Score of the H-Score)
J
FOXC2 Ab stain vs Mouse Endo genes (PDTXs)
n = 7
r² 0.256 p = 0.247n.s.
Mouse Endothelial gene expression (Z-Score)
FOXC2 antibody staining (Z-Score of the H-Score)
K
Endo genes vs FOXC2-targets (METABRIC patients)
n = 1980
r² 0.4703 p < 1x10-15
Mean FOXC2-target gene expression
Mean Endothelial gene expression
L
Colorectal Cancer (CRC) subtypes
n = 392
n = 127
CMS1/2/3
CMS4
Glioblastoma (GBM) subtypes
n = 264
n = 126
CL/PN/N
Mes
Ovarian Cancer (OVCAR) subtypes
n = 416
n = 104
Imm/Diff/Prolif
Mes
Mean FOXC2-target gene expression
M
Association of FOXC2-targets with endothelial genes across tumor types
Normalized Enrichment Score (NES)
Cell lines
Patient tumors
All FDRs < 0.001
CMS4 CRC
OVCAR
LUAD
GBM
KIRC
N
Patients with CMS4 CRC
FOXC2-targets High (n=41)
FOXC2-targets Low (n=85)
p = 0.009** HR = 1.95 (1.09-3.49)
Percent relapse-free survival
Time (Months)
Patients with Mes Ovarian Cancer
FOXC2-targets High (n=28)
FOXC2-targets Low (n=59)
p = 0.044* HR = 1.66 (0.94-2.94)
Percent disease-free survival
Time (Months)
Patients with Renal Cancer
FOXC2-targets High (n=120)
FOXC2-targets Low (n=245)
p = 0.0009*** HR = 1.85 (1.24-2.75)
Percent disease-free survival
Time (Months)

Figure S4. Related to Figure 4. FOXC2 promotes expression of endothelial genes in tumor cells of aggressive breast cancer subtypes.

(A) FOXC2 induces a distinct transcriptional program from that of traditional EMT transcription factors SNAIL, TWIST and SLUG. Correlation matrix of gene expression changes induced by over-expression of the indicated transcription factor in HMLE/R cells.

(B) Select Gene Ontology (GO) terms of genes down-regulated by FOXC2 knock down in MDA-MB-231^{VM} cells. Green (Process), Orange (Component). Shown is the -$\log_{10}$ of the BH-corrected FDR enrichment p value, dashed line indicates FDR p value of 0.05.

(C) FOXC2 knockdown in MDA-MB-231 does not significantly alter a mesenchymal gene signature. GSEA using a $\log_2$ fold change ranked list of gene expression changes with FOXC2 knockdown in MDA-MB-231 human Claudin-low breast cancer cells and Mesenchymal Gene Set#1[2]. ES = enrichment score, NES = normalized enrichment score.

(D) FOXC2 knockdown in MDA-MB-231 does not consistently alter mesenchymal marker genes. Relative mRNA levels of mesenchymal markers CDH2 (N-Cadherin) and FN1 (Fibronectin) in MDA-MB-231 cells. Bars represent mean $\log_2$ fold change (+/- SEM) vs control (shREN) extracted from DESeq2 analysis of our RNA-Seq data. **** $p < 0.0001$, DESeq2 adjusted p value, n=3 biological replicates.

(E) Heatmap representing the $\log_2$ fold change of our core FOXC2-target genes (FOXC2-target Gene Set#3) with FOXC2 knockdown in MDA-MB-231^{VM} cells, FOXC2 over-expression in HMLER cells, mouse VM clones 4T1-E^{VM} and 4T1-T^{VM} vs all clones, lung metastases-derived cell lines vs primary tumor derived cell lines from the 4T1 model and VM-proficient human cell lines vs all other solid tumor cell lines. The signature was derived as follows: We overlaid genes that were significantly up-regulated in HMLER cells with FOXC2 over-expression and significantly down-regulated with FOXC2 knockdown in MDA-MB-231 cells and then required that they show a $\log_2$ fold change >0.5 in the following comparisons: lung_vs_primary (mouse 4T1) and VM_vs_All (CCLE human) and were highly significantly (FDR $< 1 \times 10^{-5}$) up-regulated in VM 4T1 clones vs all other clones. We then ranked genes meeting those criteria by their $\log_2$ fold change in the 4T1 (VM vs all other clones) comparison and took the top 5 as our high confidence VM FOXC2-target genes.

(**F**) Protein expression levels of Endo Gene Set1 in human breast cancer cell lines, from the CCLE proteomics data, stratified by molecular subtype. Data expressed as box plots according to the Tukey convention. Mean expression of the signature was calculated for each cell line, n represents an individual cell line. *$p < 0.05$, Wilcoxon rank-sum.

(**G**) FOXC2 antibody staining of PDTX tumor sections from the indicated models. Images were analyzed using the HALO software to derive an H-Score per model. Scale bar = 50μm.

(**H**) Correlation of FOXC2 antibody H-Score vs human expression of FOXC2-target Gene Set#3 across 7 PDTX models stained in G. n represents an individual model. Pearson correlation $r^2 = 0.902$, ** $p<0.0011$, n=7.

(**I**) Correlation of FOXC2 antibody H-Score vs human expression of Endo Gene Set#1 across 7 PDTX models stained in G. n represents an individual model. Pearson correlation $r^2 = 0.932$, *** $p<0.0004$, n=7.

(**J**) Correlation of FOXC2 antibody H-Score vs mouse expression of Endo Gene Set#1 across 7 PDTX models stained in G. n represents an individual model. Pearson correlation $r^2 = 0.256$, $p = 0.247$, n=7.

(K) Correlation of FOXC2-target Gene Set#3 and Endo Gene Set#1 across breast cancer patients' tumors from the METABRIC cohort, n represents an individual patient. Pearson correlation $r^2 = 0.4703$ **** $p < 1x10^{-15}$, n=1980.

(L) FOXC2-target genes are enriched in aggressive "mesenchymal" subtypes of human colorectal, glioblastoma, and ovarian tumors. Analysis of FOXC2-target Gene Set#3 in colorectal cancer patients from the Marisa et al[3] colorectal cancer dataset, stratified by consensus molecular subtype (CMS). Glioblastoma patients from the TCGA dataset, stratified by subtype and Ovarian cancer patients from the TCGA dataset, stratified by subtype. Data expressed as box plots according to the Tukey convention. Mean expression of the signature was calculated for each patient, n represents an individual patient, **** $p < 0.0001$, Wilcoxon rank-sum.

(M) Association of FOXC2-target genes with endothelial gene expression across tumor types. GSEA summary statistics for cell lines or tumors. For each tumor type correlations of each gene with the mean expression of FOXC2-target Gene Set#1 was calculated across cell lines or across patients of that tumor type and used as a ranking metric for GSEA. Shown are the data for Endo Gene Set#1. NES = normalized enrichment score. CMS4 CRC = the CMS4 subtype of colorectal cancer, OVCAR = ovarian cancer, LUAD = lung adenocarcinoma, GBM = glioblastoma, KIRC = kidney renal carcinoma.

(N) Relapse-free survival of CMS4 colorectal cancer patients, from the Marisa et al., dataset, stratified by FOXC2-target Gene Set#3 expression. Disease-free survival of "mesenchymal" ovarian cancer patients, from the TCGA dataset, stratified by FOXC2-target Gene Set#3 expression. Disease-free survival of renal carcinoma patients, from the TCGA dataset, stratified by FOXC2-target Gene Set#3 expression. In all cases FOXC2-target high patients were those in the top 3rd of expression for that signature and the low group were the remaining $2/3^{rds}$. HR = hazard ratio. * $p < 0.05$, ** $p < 0.01$, *** $p < 0.001$, log-rank test.

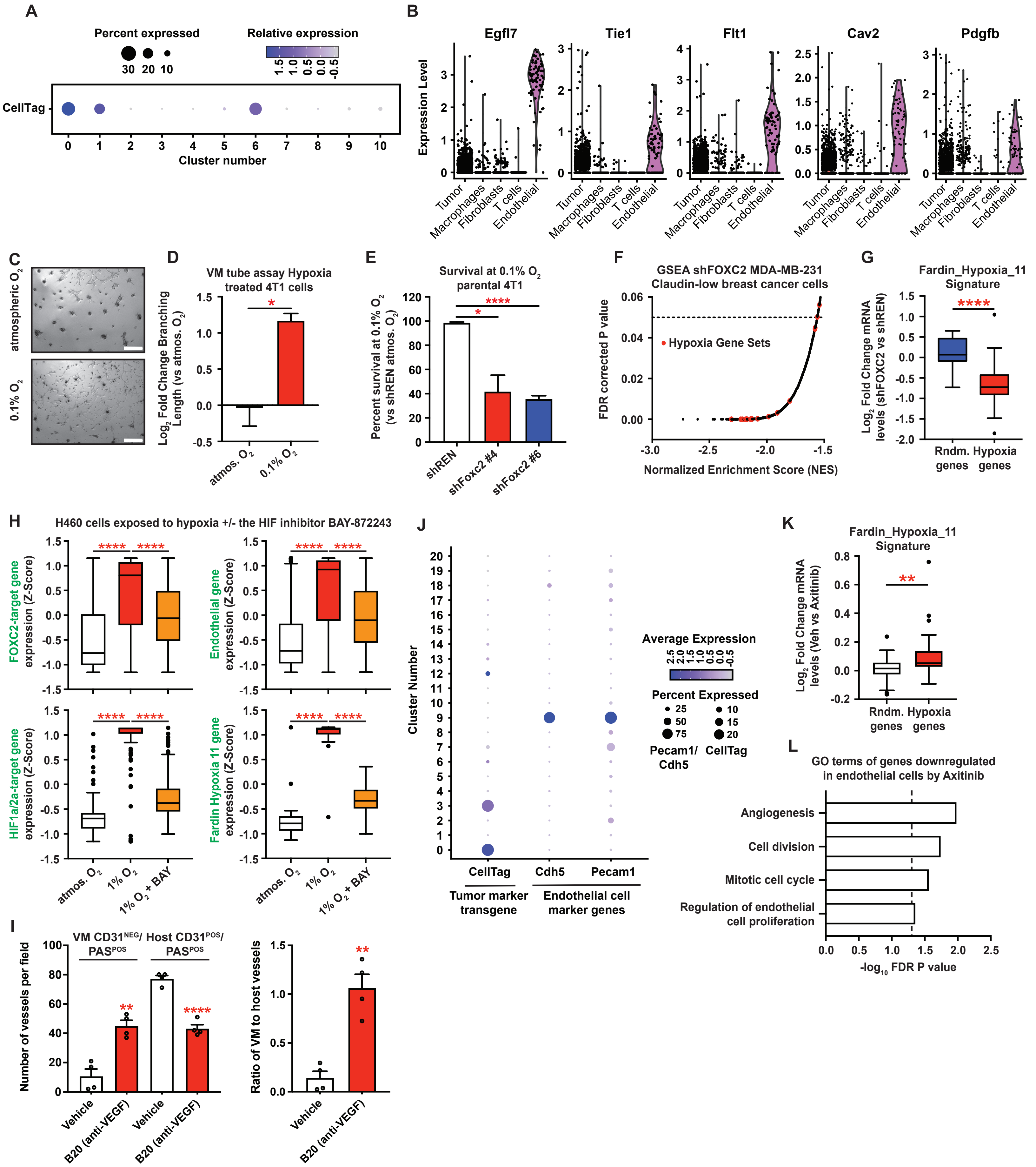

A
Percent expressed
Relative expression
CellTag
Cluster number
B
Egfl7
Tie1
Flt1
Cav2
Pdgfb
Expression Level
Tumor
Macrophages
Fibroblasts
T cells
Endothelial
C
atmospheric O2
0.1% O2
D
VM tube assay Hypoxia treated 4T1 cells
Log2 Fold Change Branching Length (vs atmos. O2)
atmos. O2
0.1% O2
E
Survival at 0.1% O2 parental 4T1
Percent survival at 0.1% O2 (vs shREN atmos. O2)
shREN
shFoxc2 #4
shFoxc2 #6
F
GSEA shFOXC2 MDA-MB-231 Claudin-low breast cancer cells
FDR corrected P value
Hypoxia Gene Sets
Normalized Enrichment Score (NES)
G
Fardin_Hypoxia_11 Signature
Log2 Fold Change mRNA levels (shFOXC2 vs shREN)
Rndm. genes
Hypoxia genes
H
H460 cells exposed to hypoxia +/- the HIF inhibitor BAY-872243
FOXC2-target gene expression (Z-Score)
Endothelial gene expression (Z-Score)
HIF1a/2a-target gene expression (Z-Score)
Fardin Hypoxia 11 gene expression (Z-Score)
atmos. O2
1% O2
1% O2 + BAY
J
Cluster Number
Average Expression
Percent Expressed
Pecam1/ Cdh5
CellTag
CellTag
Cdh5
Pecam1
Tumor marker transgene
Endothelial cell marker genes
K
Fardin_Hypoxia_11 Signature
Log2 Fold Change mRNA levels (Veh vs Axitinib)
Rndm. genes
Hypoxia genes
L
GO terms of genes downregulated in endothelial cells by Axitinib
Angiogenesis
Cell division
Mitotic cell cycle
Regulation of endothelial cell proliferation
-log10 FDR P value
I
VM CD31NEG/ PASPOS
Host CD31POS/ PASPOS
Number of vessels per field
Vehicle
B20 (anti-VEGF)
Ratio of VM to host vessels

Figure S5. Related to Figure 5. Severe hypoxia promotes quasi-endothelial differentiation of tumor cells.

(A) Dot plot representing the percentage of cells expressing, and mean expression of, CellTag in Seurat clusters from the whole scRNA-Seq dataset. Clusters 0, 1 and 6 showed greater than 20% of cells expressing CellTag and were designated tumor cells for further clustering.

(B) Violin plots from single cell RNA-Seq data from 4T1-T tumors of select "classical" endothelial genes that are expressed in a subset of 4T1-T tumor cells. Cell types were determined based on canonical markers and then grouped by broad cell type.

(C) Matrigel network formation assays of parental 4T1 cells or those same cells exposed to severe hypoxia (0.1% O_2) for 24 hrs followed by 24 hrs recovery at atmospheric O_2 levels prior to replating for tube assays. Scale bar = 500 μm.

(D) Quantification of data from C. Bars represent mean $\log_2$ fold change (+/- SEM) in branching length vs parental 4T1 at atmospheric O_2 levels. n = 3. * < 0.05, student's t-test.

(E) Survival of parental 4T1 cells expressing shREN, shFoxc2 #4, or shFOXC2 #6 under severe hypoxia (0.1% O_2) for 72 hrs followed by measuring viability by CellTiterGlo. Bars represent percent survival (+/- SEM) at 0.1% O_2 vs shREN normalized to viability at atmospheric O_2, n = 3 biological replicates. * $p < 0.05$, **** $p < 0.0001$, student's t-test.

(F) Elbow plot showing enrichment of various hypoxia-related gene sets from the msigDB "C2_all_V7.2" collection in genes down regulated in MDA-MB-231 cells with FOXC2 knockdown.

(G) Expression of the Fardin_Hypoxia_11 (genes consistently upregulated by hypoxia across 11 neuroblastoma cell lines) gene set upon FOXC2 knockdown in MDA-MB-231^{VM} cells. Data expressed as box plots according to the Tukey convention. Random genes = gene set size-matched randomly selected genes. **** $p < 0.0001$, Wilcoxon rank-sum.

(H) FOXC2-target genes and endothelial genes are induced in H460 cells in a HIF-dependent manner. Analysis of data from GSE42791 of H460 cells exposed to hypoxia (1% O_2) or hypoxia plus the HIF inhibitor BAY-872243 for FOXC2-target Gene Set#2 (375 genes), Endo Gene Set#1 (230 genes) and the mSigDB gene sets Elvidge_HIF1a_HIF2a_Targets_Dn (99 genes) and Fardin_Hypoxia_11 (32 genes). A Z-score was calculated per gene across conditions and all genes for a given signature are plotted as box plots according to the Tukey convention. **** $p < 0.0001$, Wilcoxon rank-sum.

(I) Alternative quantification of CD31/PAS staining shown in Figure 5D. Bar plots showing number of VM vessels (CD31 negative/PAS positive) and host vessels (CD31 positive/PAS positive) with or without B20 treatment (Left panel). Right panel shows the ratio of VM to host vessels. Bars represent mean number of channels (+/- SEM) per field, n = 4 fields per condition from two different animals, ** $p < 0.01$, **** $p < 0.0001$, student's t-test.

(J) Dot plot representing the percentage of cells expressing, and mean expression of, endothelial markers Pecam1 (aka CD31) and Cdh5 (aka VE-Cadherin) and the tumor marker CellTag in Seurat clusters from the whole parental 4T1 Axitinib scRNA-Seq dataset. Cluster 9 cells show strong expression of Pecam1 and Cdh5 so were designated as endothelial cells.

Clusters 0 and 3 showed greater than 20% of cells expressing CellTag and were designated tumor cells for further clustering.

(K) Confirmation that Axitinib induces hypoxia in parental 4T1 CellTag tumors. Pseudo-bulk analysis of tumour cells (Clusters 0 and 3) plus or minus Axitinib for the Fardin_Hypoxia_11 signature. ** $p < 0.01$, Wilcoxon rank-sum.

(L) Select GO term enrichment of genes significantly down regulated in endothelial cells (Cluster 9) demonstrate suppression of angiogenesis and endothelial cell proliferation in response to Axitinib.

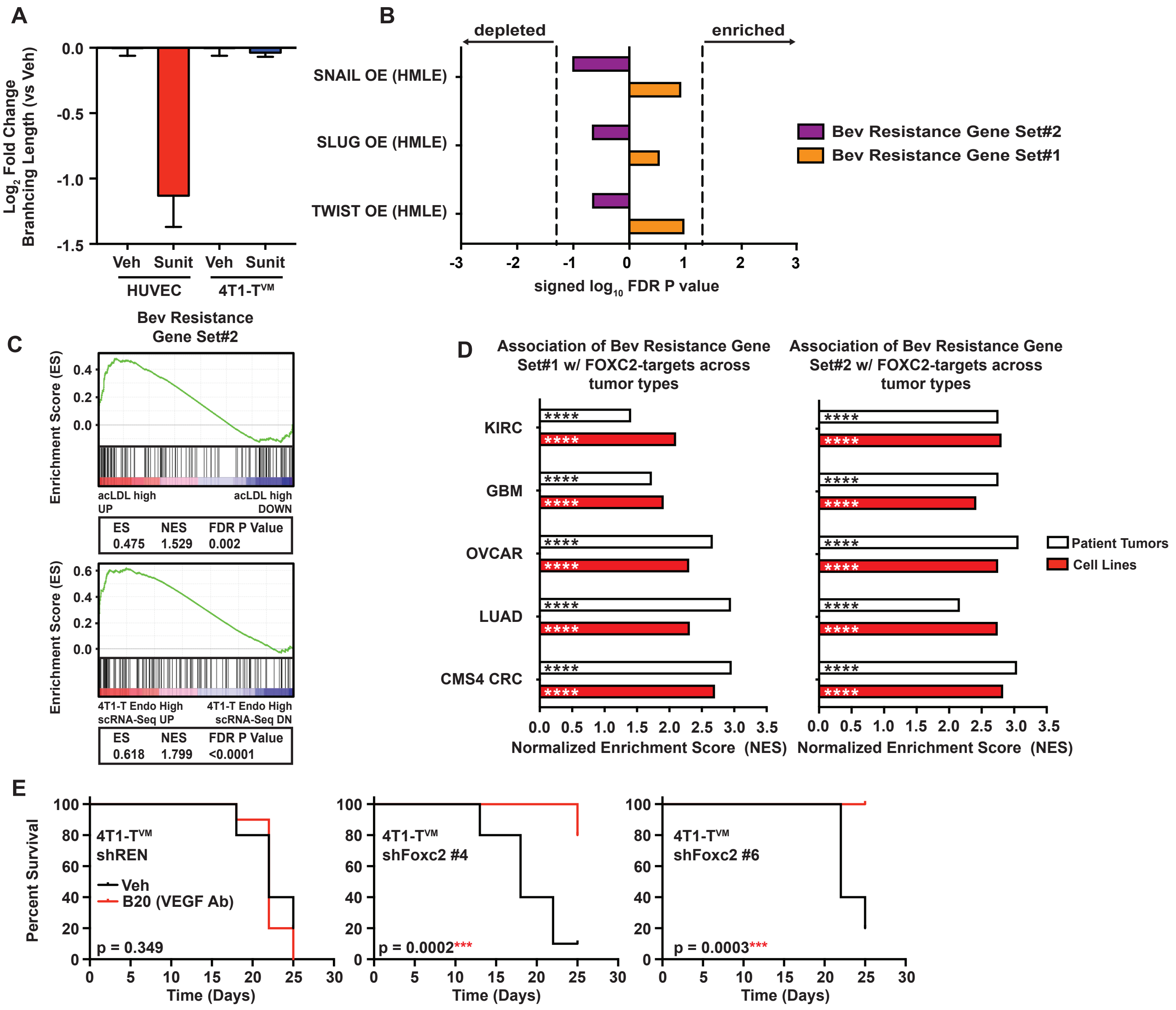

A
Log2 Fold Change Branhcing Length (vs Veh)
0.0
-0.5
-1.0
-1.5
Veh
Sunit
Veh
Sunit
HUVEC
4T1-TVM
B
depleted
enriched
SNAIL OE (HMLE)
SLUG OE (HMLE)
TWIST OE (HMLE)
-3
-2
-1
0
1
2
3
signed log10 FDR P value
Bev Resistance Gene Set#2
Bev Resistance Gene Set#1
Bev Resistance Gene Set#2
C
Enrichment Score (ES)
0.4
0.2
0.0
acLDL high UP
acLDL high DOWN
ES 0.475
NES 1.529
FDR P Value 0.002
0.6
0.4
0.2
0.0
4T1-T Endo High scRNA-Seq UP
4T1-T Endo High scRNA-Seq DN
ES 0.618
NES 1.799
FDR P Value <0.0001
D
Association of Bev Resistance Gene Set#1 w/ FOXC2-targets across tumor types
Association of Bev Resistance Gene Set#2 w/ FOXC2-targets across tumor types
KIRC
GBM
OVCAR
LUAD
CMS4 CRC
****
Patient Tumors
Cell Lines
0.0
0.5
1.0
1.5
2.0
2.5
3.0
3.5
Normalized Enrichment Score (NES)
E
Percent Survival
100
80
60
40
20
0
4T1-TVM shREN
Veh
B20 (VEGF Ab)
p = 0.349
4T1-TVM shFoxc2 #4
p = 0.0002***
4T1-TVM shFoxc2 #6
p = 0.0003***
0
5
10
15
20
25
30
Time (Days)

Figure S6. Related to Figure 6. FOXC2-driven VM promotes resistance to anti-angiogenic therapy.

(A) VM network formation is indifferent to inhibition of the VEGF receptor. Quantification of Matrigel network formation assays of HUVEC endothelial cells and 4T1-T^{VM} cells treated with Sunitinib, a multi-RTK inhibitor that targets VEGFR2. Data are represented as mean $\log_2$ fold change (+/- SEM) in branching length relative to vehicle treated cells.

(B) Lack of enrichment of AAT resistance gene sets (for gene set details see supplementary table 1) with over-expression of EMT TFs in HMLE cells. Shown is the $\log_{10}$ FDR p value derived from GSEA signed according to whether genes are depleted (negative values) or enriched (positive values). Dashed line represents FDR p value of 0.05 in either direction.

(C) Anti-angiogenic therapy resistance genes from Bevacizumab resistant GBM xenografts (Bev Resistance Gene Set#2) are upregulated in acLDL high 4T1-T^{VM} cells and endo high 4T1-T tumor cells. Analysis of RNA-seq data generated from 4T1-T^{VM} sorted based on their uptake of acLDL or of scRNA-seq data generated from CellTag 4T1-T^{VM} tumors for enrichment of Bev Resistance Gene Set#2.

(D) FOXC2-target genes correlate with AAT resistance genes across tumor types. GSEA summary statistics for cell lines or tumors. For each tumor type correlation of each gene with the mean expression FOXC2-target Gene Set#3 was calculated across cell lines or patients within that tumor type. These correlation coefficients were used as a ranking metric for GSEA. Shown are the results for the gene set consisting of genes that are upregulated in breast cancer patients that fail to respond to Bevacizumab (Bev Resistance Gene Set#1, left panel) or genes that are upregulated upon Bevacizumab resistance in a U87-MG Glioblastoma xeongraft model (right panel, Bev Resistance Gene Set#2, GSE37956). NES = normalized enrichment score. **** $p < 0.0001$, GSEA-derived FDR. CMS4 CRC = the CMS subtype of colorectal cancer, LUAD = lung adenocarcinoma, OVCAR = ovarian cancer, GBM = Glioblastoma, KIRC = kidney renal carcinoma.

(E) Suppression of Foxc2 increases survival in response to AAT in the 4T1 model. Survival curves of mice implanted with control (shREN) or Foxc2 shRNA expressing 4T1-T^{VM} tumors cells +/- B20 treatment, *** $p < 0.001$, log-rank test. Time represents the time, in days, from implantation to point of 20% body weight loss or 250 mm^3 tumor volume.

Supplementary References

1. Pearsall, S.M., Williamson, S.C., Marqués, F.J.G., Humphrey, S., Hughes, E., Shue, Y.T., Bermudez, A., Frese, K.K., Galvin, M., Carter, M., et al. (2022). Lineage plasticity in SCLC generates non-neuroendocrine cells primed for vasculogenic mimicry. Biorxiv, 2022.10.21.512986. 10.1101/2022.10.21.512986.

2. Evrard, S.M., Lecce, L., Michelis, K.C., Nomura-Kitabayashi, A., Pandey, G., Purushothaman, K.-R., d'Escamard, V., Li, J.R., Hadri, L., Fujitani, K., et al. (2016). Endothelial to mesenchymal transition is common in atherosclerotic lesions and is associated with plaque instability. Nat Commun *7*, 11853. 10.1038/ncomms11853.

3. Marisa, L., Reyniès, A. de, Duval, A., Selves, J., Gaub, M.P., Vescovo, L., Etienne-Grimaldi, M.-C., Schiappa, R., Guenot, D., Ayadi, M., et al. (2013). Gene Expression Classification of Colon Cancer into Molecular Subtypes: Characterization, Validation, and Prognostic Value. Plos Med *10*, e1001453. 10.1371/journal.pmed.1001453.
